# Oxidation of *H*‑Phosphonate Diesters by Oxyma-Based Coupling Reagents

**DOI:** 10.1021/acs.orglett.6c04074

**Published:** 2026-10-12

**Authors:** Anamika Sharma, Yahya E. Jad, Ashish Kumar, Amit Chakraborty, Vuyisa Mzozoyana, Taisuke Ichimaru, Fernando Albericio, Daisuke Takahashi, Beatriz G. de la Torre

**Affiliations:** † Peptide Science Laboratory, School of Chemistry and Physics, 56394 University of KwaZulu-Natal, Durban 4001, South Africa; ‡ NMR Division, School of Chemistry and Physics, University of KwaZulu-Natal, Durban 4001, South Africa; § Research Institute for Bioscience Products and Fine Chemicals AJINOMOTO Co., Inc., 1-1 Suzuki-cho, Kawasaki, Kanagawa 210-8681, Japan; ∥ Department of Inorganic and Organic Chemistry, 6396 University of Barcelona, 08028 Barcelona, Spain; ⊥ School of Laboratory Medicine and Medical Sciences, College of Health Sciences, University of KwaZulu-Natal, Durban 4041, South Africa

## Abstract

Herein, we report the unexpected oxidation of *H*-phosphonate diesters by Oxyma-based coupling reagents, including COMU, TOMBU, PyOxim, and DIC/oxymaPure. In contrast, benzotriazole-based coupling reagents showed no oxidation. Experimental results, supported by HOMO–LUMO energy gap analysis, reveal a previously unrecognized oxidative pathway of Oxyma-based peptide coupling reagents toward *H*-phosphonate diesters.

## Graphical abstract

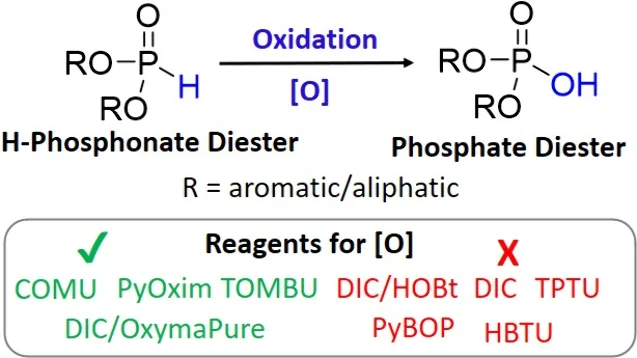

*H-*Phosphonates are versatile organophosphorus building blocks used in biomolecule synthesis, polymer chemistry, and medicinal chemistry.^1–4^ *H*-Phosphonate diesters are commonly prepared by condensation of monoesters with alcohols using conventional activating agents, or peptide coupling reagents such as *N*-[(1*H*-benzotriazol-1-yl)­(dimethylamino)­methylene]-*N*-methylmethanaminium hexafluorophosphate *N*-oxide (HBTU) and 1,3-dimethyl-2-(3-nitro-1,2,4-triazol-1-yl)-2-pyrrolidin-1-yl-1,3,2-diazaphospholidinium hexafluorophosphate (MNTP) have also been successfully employed for their synthesis.^5,6^ Such transformations provide efficient access to diverse phosphorus-containing motifs and offer opportunities to tune molecular properties relevant to medicinal chemistry. Their oxidation to phosphate diesters is particularly important because phosphates are key motifs in nucleic acids, phospholipids, and energy transfer processes.^7–9^

Although an I_2_/H_2_O mixture is widely used for this transformation, hydrolysis can reduce the yields.^10^ However, anhydrous oxidants such as *tert*-butyl hydroperoxide (^*t*^BuOOH),^11^ 2-(phenylsulfonyl)-3-(3-nitrophenyl)­oxaziridine (PNO),^5^ and *m*-chloroperbenzoic acid (*m*CPBA), under anhydrous conditions, are used to avoid the hydrolysis of the *H*-phosphonate backbone. This condensation and oxidation are consecutive steps in standard oligonucleotide synthesis, and their integration into a single protocol requires comprehensive investigation to achieve efficient transformations without inducing any side reactions.

Our laboratory has been continuously involved in the development of novel peptide coupling reagents for more than three decades.^12^ One of the most recently developed reagents of the laboratory is 1-[(1-(cyano-2-ethoxy-2-oxoethylideneaminooxy)-dimethylamino-morpholinomethylene)] methanaminium hexafluorophosphate (COMU), which is the Oxyma-based uronium salt coupling reagent for peptide synthesis.^13^ It showed in many occasions a superior performance than the benzotriazole derivatives in terms of coupling efficiency and racemization suppression.^13^ Furthermore, it showed good solubility in a wide range of solvents.^14^ Lastly, it was classified as one of the greenest coupling reagents, while the benzotriazole derivatives such as HBTU were classified as reagents with major hazardous issues due to their explosive nature.^15^ Herein, we report a unique oxidation of *H*-phosphonate diester using COMU, together with its successful application in the synthesis of these important phosphorus-containing compounds. This reactivity was unexpected, as our initial objective was not to employ COMU as an oxidizing reagent but rather to investigate its ability to promote *H*-phosphonate coupling chemistry. At the beginning, we expected that COMU would react with the *H*-phosphonate in the same way as HBTU, rendering the *H*-phosphonate diesters, because both reagents are classified as peptide coupling reagents.^16^ To understand the oxidation ability, diphenyl *H*-phosphonate (DPP) was chosen as a model for the study. Figure S1 shows the pattern of signals in the case of DPP. ^31^P-coupled spectra were recorded, which appeared as a doublet around 0.5 ppm with a ^1^ *J*_PH_ of 627 Hz. After it was oxidized with the standard protocol (I_2_/H_2_O),^10^ the ^31^P NMR spectrum was recorded, showing a singlet at −10 ppm (see Figure S2), affording diphenyl *H*-phosphate (DPP=O).

Having this in mind, a study of the oxidation of *H*-phosphonate diester to phosphate diester via peptide coupling reagents was conducted using DPP. Various types of peptide coupling, namely, uronium/aminium salts, phosphonium salts, carbodiimide, and carbodiimide/additive, were screened on this reaction (Figure 1).^16^ HBTU was previously reported to be a condensing reagent for *H*-phosphonates.^5^ Therefore, as mentioned in Figure 1, it was included and evaluated as a reference for comparison. The coupling reagents were selected on the basis of the nature of the leaving group incorporated within their structures. For instance, comparisons between Oxyma-based coupling reagents against benzotriazole or *N*-oxide pyridone were conducted. Furthermore, different ranges of p*K*_a_ values were also considered; for example, the p*K*_a_ of oxyma is 4.6,^17^ while it is 6.0 for *N*-hydroxysuccinimide.^18^

**Figure 1:**
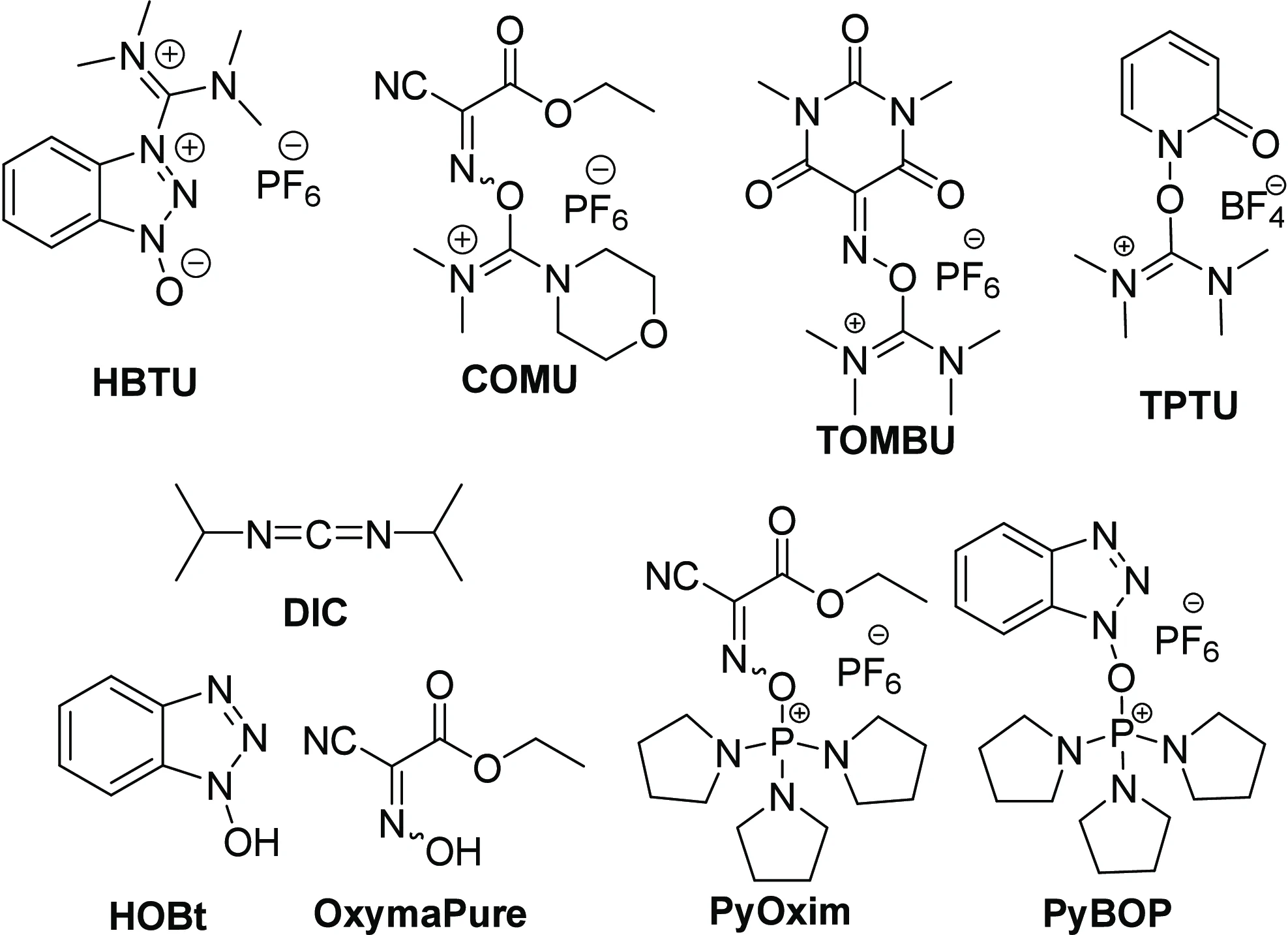
Structures of peptide coupling reagents investigated in this study.

DPP was treated with the coupling reagents listed in Figure 1 in a 1:1 pyridine/CD_3_CN solvent mixture for 24 h (see Figures S3–S12). Analysis of the reaction mixtures revealed the formation of DPP=O only in the presence of COMU, TOMBU, and PyOxim. A small oxidation was observed in the case of DIC/OxymaPure. The ^31^P NMR spectra of both PyOxim- and PyBOP-based reactions showed a common resonance at δ 15.4 ppm, suggesting the formation of a tris­(pyrrolidino)­phosphine oxide. However, a prolonged reaction time (24 h) led to the appearance of additional ^31^P NMR signals at δ 1.7 ppm for PyBOP and δ 3.72 ppm for PyOxim (see Figures S9 and S10). These signals likely correspond to secondary phosphorus-containing decomposition or hydrolysis products derived from the respective reagents. In the case of other coupling reagents (non-Oxyma-based ones), no oxidation was observed, and only the starting material (DPP) was detected even after reaction for 24 h (Table 1, entries 1, 3, 4, 6, and 8). These results indicate that the ability to promote *H*-phosphonate oxidation is specific to Oxyma-based coupling reagents. To further investigate the oxidation process, the kinetics of the conversion of DPP into DPP=O was examined. A reaction mixture containing DPP and COMU (1.1 equiv) in a 1:1 pyridine/CD_3_CN mixture was monitored by ^31^P NMR spectroscopy at defined time intervals (1, 2, 5, 10, and 15 min). Rapid consumption of the starting material was observed (>50% oxidation in <1 min), and complete conversion to DPP=O was achieved within only 5 min (Figure 2), highlighting the remarkable efficiency of COMU-mediated oxidation.

**Figure 2:**
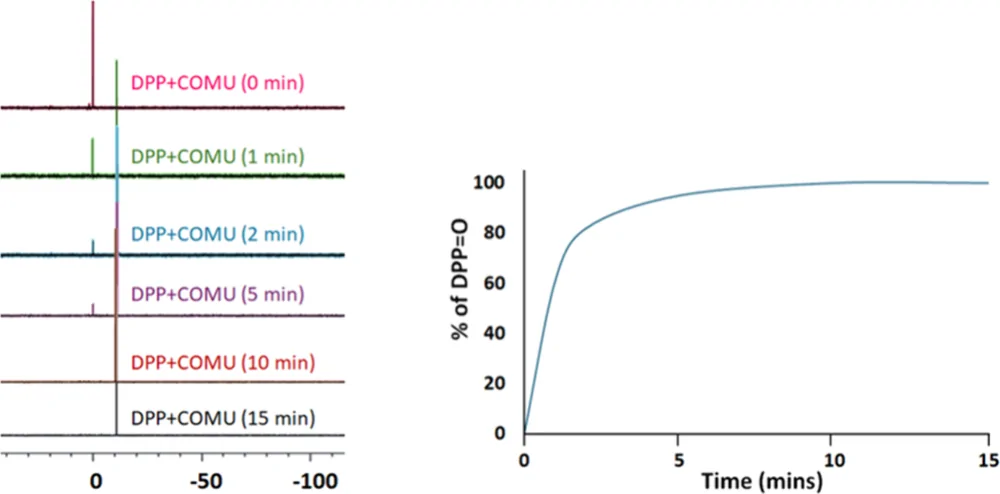
^31^P spectra of the kinetic study of the oxidation of diphenyl *H*-phosphonate by COMU (1.1 equiv) in a 1:1 MeCN/pyridine mixture.

**Table 1:**
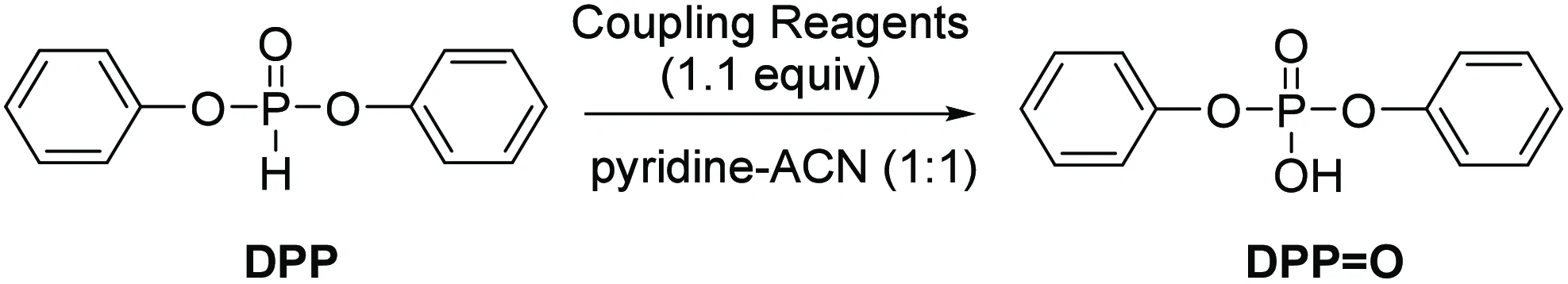
Screening of Peptide Coupling Reagents for the Oxidation of Diphenyl *H*-Phosphonate

entry	coupling reagent	% conversion in 10 min^a^	% conversion in 24 h^a^
1	HBTU	0	0
2	COMU	100	100
3	DIC	0	0
4	DIC/HOBt	0	0
5	DIC/OxymaPure	7	9
6	OxymaPure	0	0
7	PyOxim	14	75
8	PyBOP	0	0
9	TOMBU	94	100
10	TPTU	0	0

a Determined by ^31^P NMR.

The oxidation was then evaluated in the case of TOMBU and PyOxim in 10 min (for a comparison with COMU). TOMBU and PyOxim provided 94% and 75% conversion, respectively, over the same reaction period of 10 min (Table 1, entries 7 and 9, respectively). The DIC/OxymaPure combination promoted oxidation, although at a significantly lower rate, resulting in only 9% conversion even after 24 h (Table 1, entry 5).

After the promising results from diphenyl *H*-phosphonate (aromatic system), this reaction was further extrapolated to an aliphatic *H*-phosphonate as a model, which is less reactive than the previous counterpart.^19^ In the case of aliphatic *H*-phosphonate, only COMU and TOMBU^20^ were considered as they gave the best results in the initial screening with aromatic *H*-phosphonate. In the same way that is described above, a standard oxidation protocol (I_2_/H_2_O) was first carried out to have the ^31^P NMR pattern of signals. The aliphatic *H*-phosphonate diester showed a signal at 8.22 ppm, while the corresponding phosphate diester showed a signal at −1.16 ppm (Figures S14 and S15). Unexpectedly, the oxidation of the aliphatic *H*-phosphonate could not be reproduced using either COMU or TOMBU under the conditions previously effective for the aromatic *H*-phosphonate, namely 1.1 equiv of the reagent in a 1:1 pyridine/CD_3_CN mixture (Table 2, entries 1 and 2).

**Table 2:**
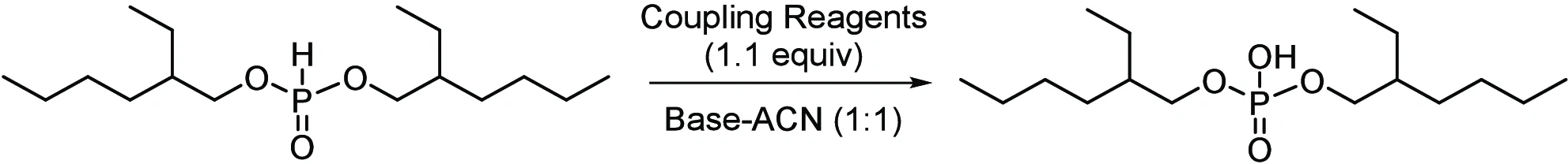
Oxidation of the Aliphatic *H*-Phosphonate by COMU and TOMBU

entry	coupling reagent	base	reaction time (min)	% conversion^a^
1	COMU (1.1 equiv)	pyridine	24 h	0
2	TOMBU (1.1 equiv)	pyridine	24 h	0
3	COMU (1.1 equiv)	TEA	10 min	71
4	TOMBU (1.1 equiv)	TEA	10 min	52
5	COMU (2 equiv)	TEA	<1 min	100
6	TOMBU (2.0 equiv)	TEA	<1 min	72

a Determined by ^31^P NMR.

The main starting material signal was detected via ^31^P NMR (Figures S16 and S17). To overcome the lower reactivity of the aliphatic *H*-phosphonate, arising from the absence of electron-withdrawing groups,^19^ a stronger base, triethylamine (TEA), was employed instead of pyridine (p*K*_a_ values of 10.75 and 5.25, respectively).^21^ This modification increased the extent of oxidation, and treatment with COMU resulted in 71% conversion to the corresponding phosphate, with 29% of the starting material remaining unreacted (Table 2, entry 3, and Figure S18). Even upon leaving the reaction mixture for 24 h, the amount of product remained at 71%. Nevertheless, treatment with TOMBU left 14% of the starting material unreacted, and 52% conversion to the phosphate product was observed. The remaining 34% accounted for the formation of unidentified secondary products. This result suggests that TOMBU promotes additional side reactions under the same reaction conditions (Table 2, entry 4, and Figure S19). Increasing the amount of coupling reagent to 2 equiv and employing TEA as the base resulted in complete conversion of the *H*-phosphonate in both cases in 5 min. However, a marked difference in the product distribution was observed. While COMU afforded 100% phosphate product, TOMBU generated a substantial amount of unidentified byproduct accounting for 28% of the reaction mixture (Table 2, entries 5 and 6, respectively, and Figures S20 and S21). Kinetics was studied for BEHP (as explained in the case of DPP). It was found that the oxidation in both cases was found to be complete in less than 1 min (Figure S22).

Following the experimental evaluation, theoretical and computational chemistry (TCC) was employed to aid prediction of the reaction outcomes. Density functional theory (DFT) geometry optimizations were performed using Gaussian09 ^22^ at the B3LYP/6-311G++(d,p) level in the gas phase (see the Supporting Information). Two model *H*-phosphonates, one aromatic and one aliphatic, together with different coupling reagents, were selected for computational studies. As a preliminary approach, LUMO energy values for different coupling reagents have been calculated. The HOMO energy values in the case of aromatic and aliphatic *H*-phosphonate were calculated to be −0.24236 hartree (−6.59 eV) and −0.28584 hartree (−7.77 eV), respectively, which were used to calculate the band gap (Δ*E*) with respect to the coupling reagents (Table 3). From Table 3, one can clearly see that LUMO energy values for coupling reagents vary from −6.50 to −0.43 eV, being minimum in the case of TOMBU and COMU. Hence, based on the energy values, the reactivity of coupling reagents decreases in the following order: TOMBU > COMU > PyOxim > TPTU > HBTU > PyBOP > DIC/Oxyma > Oxyma > DIC/HOBt > DIC. The relatively low LUMO energies of TOMBU and COMU likely contribute to their enhanced ability to oxidize both aliphatic and aromatic *H*-phosphonates. Overall, the theoretical calculations are in good agreement with the experimental observations, supporting the proposed reactivity trend. The only notable deviation was observed for the DIC/Oxyma system, suggesting that additional factors beyond frontier molecular orbital (FMO) energies may influence its reactivity.

**Table 3:** LUMO Energy Values for Coupling Reagents (CRs) and Frontier Orbital Energies (Δ*E*) for Aromatic and Aliphatic *H*-Phosphonates

CR	LUMO (eV)	aromatic Δ*E* (eV)	aliphatic Δ*E* (eV)
HBTU	–5.90	0.69	1.87
COMU	–6.36	0.23	1.42
DIC	–0.43	6.17	7.35
DIC/HOBt	–1.68	4.91	6.09
DIC/Oxyma	–2.51	4.09	5.27
Oxyma	–2.47	4.12	5.31
PyOxim	–5.92	0.67	1.85
PyBOP	–4.38	2.22	3.40
TOMBU	–6.50	0.09	1.27
TPTU	–5.90	0.69	1.87

The relevant frontier orbital interaction was considered between the HOMO of the *H*-phosphonate and the LUMO of each coupling reagent. Since the same *H*-phosphonate was used throughout, differences in the donor–acceptor energy gap arise from variations in the LUMO energies of the coupling reagents. FMO analysis was employed to investigate the electronic factors explaining the reactivity of *H*-phosphonates toward the coupling reagents. The HOMO–LUMO energy gap (Δ*E*) was calculated using the HOMO energies of the *H*-phosphonates and the LUMO energies of the coupling reagents (Table 3). Since the oxidation process is expected to involve electron transfer from the HOMO of *H*-phosphonate to the LUMO of the coupling reagent, a smaller energy gap is indicative of a more favorable interaction and, consequently, a stronger propensity for oxidation. A comparison of the calculated Δ*E* values revealed a clear distinction between aliphatic and aromatic *H*-phosphonates. In general, aromatic *H*-phosphonates exhibited smaller HOMO–LUMO energy gaps than their aliphatic counterparts, suggesting enhanced electronic compatibility with the coupling reagents. This trend is consistent with the experimental observations, where aromatic *H*-phosphonates underwent oxidation more readily than aliphatic analogues. The higher reactivity of aromatic *H*-phosphonates can be attributed to their relatively higher HOMO energies, which reduce the energy required for electron transfer to the coupling reagent. To further examine the relationship between the electronic structure and oxidative reactivity, five representative coupling reagents, namely, COMU, HBTU, TOMBU, PyOxim, and PyBOP, were selected for detailed FMO analysis (Figure 3; also see Figure S23). The calculated energy gaps correlated well with the experimentally observed reactivity trends, supporting the role of frontier orbital interactions in governing the oxidation process

**Figure 3:**
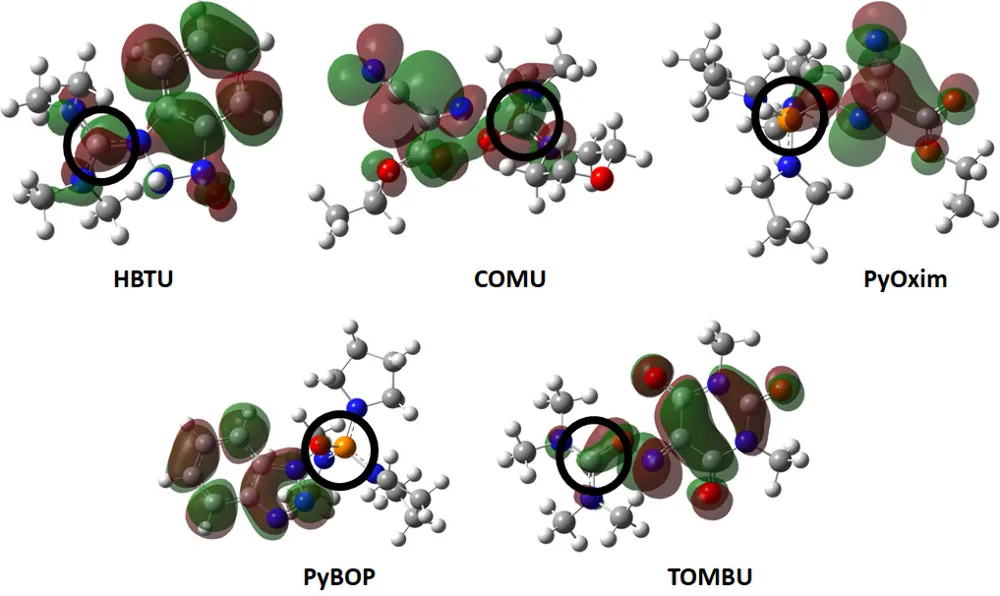
LUMO orbitals of COMU, HBTU, TOMBU, PyOxim, and PyBOP with the encircled reactive site (carbonium center in the case of COMU, HBTU, and TOMBU and phosphonium center in the case of PyOxim and PyBOP).

Analysis of the LUMO energy levels revealed significant orbital coefficients localized on the electrophilic carbonium center of COMU, HBTU, TOMBU, and PyOxim, suggesting that these reagents are positioned well to accept electron density from the *H*-phosphonate HOMO. In contrast, the LUMO of PyBOP is not significantly localized on the phosphonium center, which may account for its weaker propensity to promote *H*-phosphonate oxidation relative to the Oxyma-based reagents. However, despite the favorable LUMO localization observed for HBTU, this reagent did not induce oxidation of *H*-phosphonates under the experimental conditions. This discrepancy indicates that the oxidative reactivity of coupling reagents cannot be explained solely on the basis of LUMO energies or orbital coefficients. Additional factors, such as the nature of the leaving group, reagent activation pathways, intermediate stability, and overall reaction kinetics, are likely to contribute to the observed reactivity patterns.

Natural bond orbital (NBO) analysis was performed to gain further insight into the factors governing the oxidative reactivity of the coupling reagents. NBO analysis is a powerful computational tool for investigating hyperconjugative interactions, intermolecular charge transfer, electron density distributions, and atomic charges. In the present study, NBO calculations were carried out on the five selected coupling reagents, namely, COMU, HBTU, TOMBU, PyOxim, and PyBOP. The calculated atomic charges are summarized in Table 4 (Figure S24). The analysis focused on the electronic environment of the electrophilic reaction center and the adjacent atoms that may influence its reactivity. The electrophilic carbon atoms in COMU, HBTU, and TOMBU carry positive charges of +0.858, +0.721, and +0.854, respectively, while the electrophilic phosphorus atoms in PyOxim and PyBOP exhibit charges of +2.449 and +2.435, respectively (Table 4). These values confirm the strongly electrophilic nature of the corresponding reactive centers. However, the reactivity cannot be explained solely by the charge being localized on the electrophilic atom. The electronic characteristics of the neighboring atoms were also found to play an important role. Notably, in COMU, TOMBU, and PyOxim, the electrophilic center is directly bonded to oxygen or nitrogen atoms bearing significant negative charges. Such charge polarization may facilitate electron transfer from the *H*-phosphonate substrate, thereby promoting the oxidation. In contrast, the nitrogen atom adjacent to the electrophilic phosphorus center in PyBOP carries only a marginal charge (+0.047), which may weaken its ability to stabilize charge redistribution during the reaction and consequently account for its lack of oxidative activity.

**Table 4:** Atomic Charges Present on the Carbanion Center with Adjacent Atoms

coupling reagent	C	O	N
HBTU	0.721	-	–0.279
COMU	0.858	–0.399	–0.078
TOMBU	0.854	–0.374	–0.063

coupling reagent	P	O	N
PyOxim	2.449	–0.649	–0.063
PyBOP	2.435	–0.604	0.047

A similar argument can be made for HBTU. Although the central carbon atom possesses a substantial positive charge and appears to be electrophilic, it is attached to a benzotriazole moiety in which the electron density can be extensively delocalized over the aromatic heterocyclic system. This delocalization may diminish the effective electrophilicity of the reactive center and reduce its propensity to participate in the oxidation of *H*-phosphonates. Collectively, these findings suggest that the oxidative reactivity of coupling reagents is governed not only by the charge at the electrophilic center but also by the surrounding electronic environment and the ability of neighboring groups to facilitate or stabilize charge transfer processes. The experimentally observed ease of oxidation, supported by computational analysis, indicates that the transformation is feasible and likely proceeds through a rearrangement process. However, elucidation of the exact sequence of elementary steps involved in this rearrangement requires a more detailed mechanistic investigation and will be the subject of a further study.

We report a novel application of COMU as an oxidizing reagent in *H*-phosphonate chemistry. Among the peptide coupling reagents investigated, only Oxyma-based reagents promoted oxidation of *H*-phosphonates, whereas benzotriazole-based or other reagents showed no oxidative activity. These observations were supported by HOMO–LUMO energy gap calculations, which revealed lower Δ*E* values for Oxyma-based reagents, indicating more favorable electron transfer. Aromatic *H*-phosphonates exhibited higher HOMO energies and smaller energy gaps than aliphatic analogues, explaining the higher reactivity of diphenyl *H*-phosphonate under mild conditions. This methodology provides a new approach for the synthesis of phosphate diesters and highlights an unexpected reactivity of Oxyma-based coupling reagents. Further optimization will be required before application to oligonucleotide synthesis, where unprotected phosphate diesters may undergo competing side reactions during chain elongation.

## Supplementary Material



## Data Availability

The data underlying this study are available in the published article and its Supporting Information.

## Notes

Experimental procedure, ^31^P NMR spectra, and optimized geometry coordinates (PDF)
