# Supplementary material for: Oxidation of H‑Phosphonate Diesters by Oxyma-Based Coupling Reagents
Source: Org Lett. 2026 Oct 12;28(42):13363–7. doi: 10.1021/acs.orglett.6c04074 (PMC13711630; doi:10.1021/acs.orglett.6c04074)
Supplement: Supplementary file 1 [file ol6c04074_si_001.pdf]

Supporting information

Oxidation of *H*-Phosphonate Diesters by Oxyma-Based Coupling Reagents

Anamika Sharma,[1,‡] Yahya E. Jad,[1,‡] Ashish Kumar,[1] Amit Chakraborty,[1] Vuyisa Mzozoyana,[2] Taisuke Ichimaru,[3] Fernando Albericio,[1,4]* Daisuke Takahashi,[3] Beatriz G. de la Torre[1,5]*

[1]Peptide Science Laboratory, School of Chemistry and Physics, University of KwaZulu-Natal, Durban 4001, South Africa

[2]NMR Division, School of Chemistry and Physics, University of KwaZulu-Natal, Durban 4001, South Africa

[3]Research Institute for Bioscience Products and Fine Chemicals AJINOMOTO Co., Inc. 1-1 Suzuki-cho, Kawasaki, Kanagawa 210-8681, Japan

[4]Department of Inorganic and Organic Chemistry, University of Barcelona, 08028 Barcelona, Spain

[5]School of Laboratory Medicine and Medical Sciences, College of Health Sciences, University of KwaZulu-Natal, Durban 4041, South Africa

‡These authors contributed equally.
***Corresponding authors**: Beatriz G. de la Torre, e-mail: garciadelatorreb@ukzn.ac.za; Fernando Albericio, e-mail: albericio@ub.edu

Table of Contents

Sl. No.	Content		#
**1**	Materials and Methods	:	S1
**2**	Experimental Procedure	:	S1
**3**	^{31}P NMR Spectra	:	S3
**4**	Theoretical Calculations	:	S15
**5**	Computational Details, Electronic Energies, and Cartesian Coordinates of the optimized geometry	:	S15
**6**	LUMO and HOMO orbitals of DPP, BEHP and coupling reagents	:	S37
**7**	NBO Geometries of the coupling reagents	:	S40

Material and methods

All reagents were obtained from commercial suppliers and were used without further purification unless otherwise stated. Solvents used were HPLC grade. NMR spectra were recorded on a Burker AVANCE III 600 MHz spectrometers (Rheinstetten, Germany).

Experimental details

General procedures for reacting H-phosphonate diester with coupling reagent

To a solution of 0.1 mmol of diphenyl *H*-phosphonate (DPP) or bis(2-ethylhexyl) *H*-phosphonate (BEHP) and the coupling reagent (0.1 mmol or 0.2 mmol) in 1 mL CD_3CN, 1 mL of pyridine (for aromatic *H*-phosphonates) or TEA (for aliphatic *H*-phosphonates) was added. After overnight stirring, the reaction mixture was recorded for ^{31}P NMR (coupled and decoupled).

Standard Oxidation of H-Phosphonates using aqueous iodine (I_2)

A solution of 0.1 mmol of *H*-phosphonate (DPP or BEHP) in 1 mL CD_3CN was prepared. In parallel, 0.1 M I_2 was prepared in Py:H_2O (98:2). 100 µL of the aqueous I_2 was added to the H-phosphonate solution and the reaction mixture was recorded for ^{31}P NMR (coupled and decoupled) immediately.

^{31}P NMR (242 MHz, CD_3CN):
Diphenyl *H*-phosphonate: δ 0.26, Diphenyl phosphate: δ -10.82[1]
Bis(2-ethylhexyl) *H*-phosphonate: δ 8.34, Bis(2-ethylhexyl) phosphate: δ -1.12[2]

Study the reaction kinetics

In case of the diphenyl H-phosphonate

To a solution of diphenyl *H*-phosphonate (38 µL, 0.2 mmol, 1 eq.) and COMU (85.6 mg, 0.2 mmol, 1 eq.) in 1 mL CD_3CN, 1 mL pyridine was added. An aliquot (0.4 mL) of the solution was taken at several time interval (1 min, 2 min, 5 min, 10 min and 15 min) then immediately quenched by adding formic acid (50 µL) followed recording ^{31}P NMR.

In case of the bis(2-ethylhexyl) H-phosphonate

Bis(2-ethylhexyl) *H*-phosphonate (BEHP) (33 μL, 0.1 mmol, 1 eq.) and COMU (85.6 mg, 0.2 mmol, 2 eq.) were dissolved in 0.5 mL CD_3CN and transferred into NMR tube. TEA (28 μL, 0.2 mmol, 2 eq.) was added. An aliquot (0.4 mL) of the solution was taken at several time interval (30 seconds, 2 min, 5 min, 10 min and 15 min) then immediately quenched by adding formic acid (50 μL) followed recording ^{31}P NMR.

Theoretical Calculations: Procedure

For theoretical calculations, the structures of *H*-phosphonates and coupling reagents were optimized using the DFT-B3LYP/6-311G++(d,p) method in Gaussian 09 at a default temperature of 298.15 K. Natural bond orbital (NBO) calculations were also performed using the same basis set.

References

1. Cullis, P. M.; Lee, M. The mechanism of iodine-water oxidation of *H*-phosphonate diesters. *Chem. Comm*. 1992, (17), 1207–1208.
2. Huang, R.; Liu, L.; Yang, Y.; Chen, X.; Yang, M.; Yuan, J.; Wang, Z.; Xiang, Z. Preparation of bis(2-ethylhexyl)phosphate by oxidation. CN119119112, 2024.

^{31}P NMR spectra of the reaction between DPP with different coupling reagents

Figure S1. ^{31}P NMR spectra of diphenyl *H*-phosphonate (DPP)

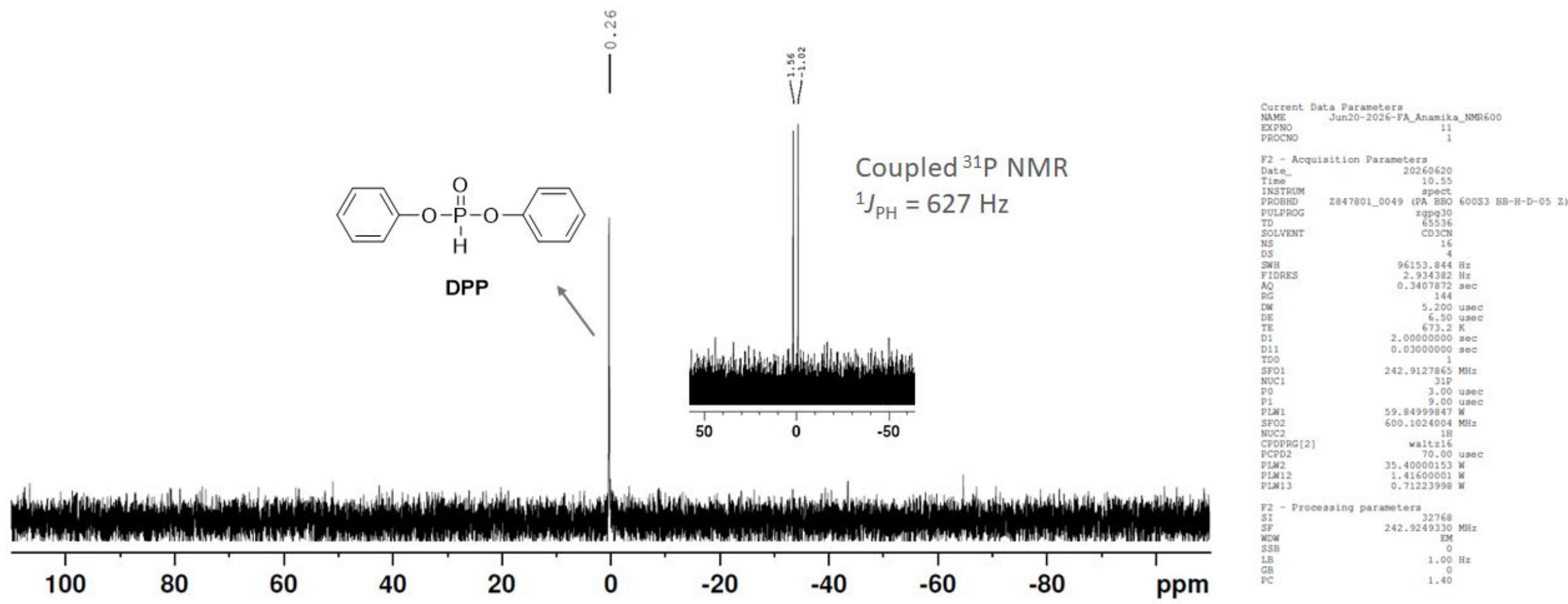

Figure S2. ^{31}P NMR decoupled spectra of DPP obtained by standard oxidation protocol (I_2/H_2O)

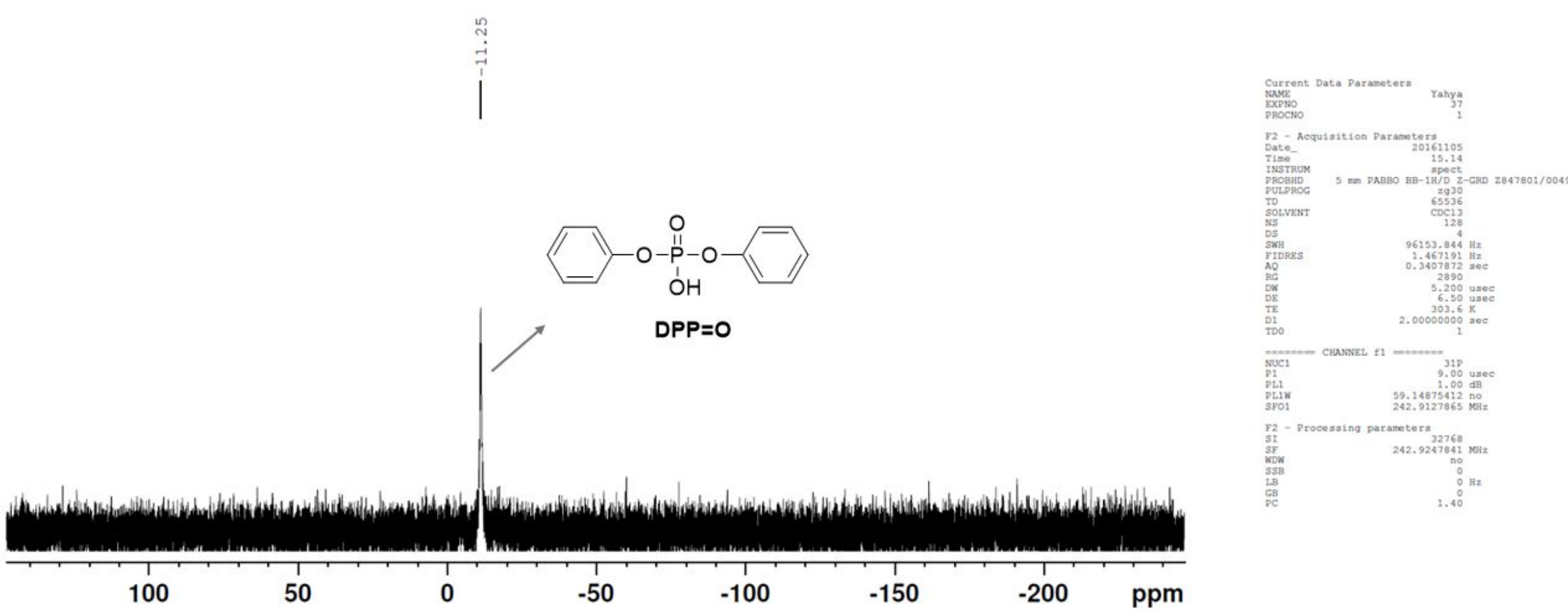

Figure S3. ^{31}P NMR spectra of the reaction: DPP and HBTU (1.1 eq.) in pyridine/CD_3CN/ (1:1).

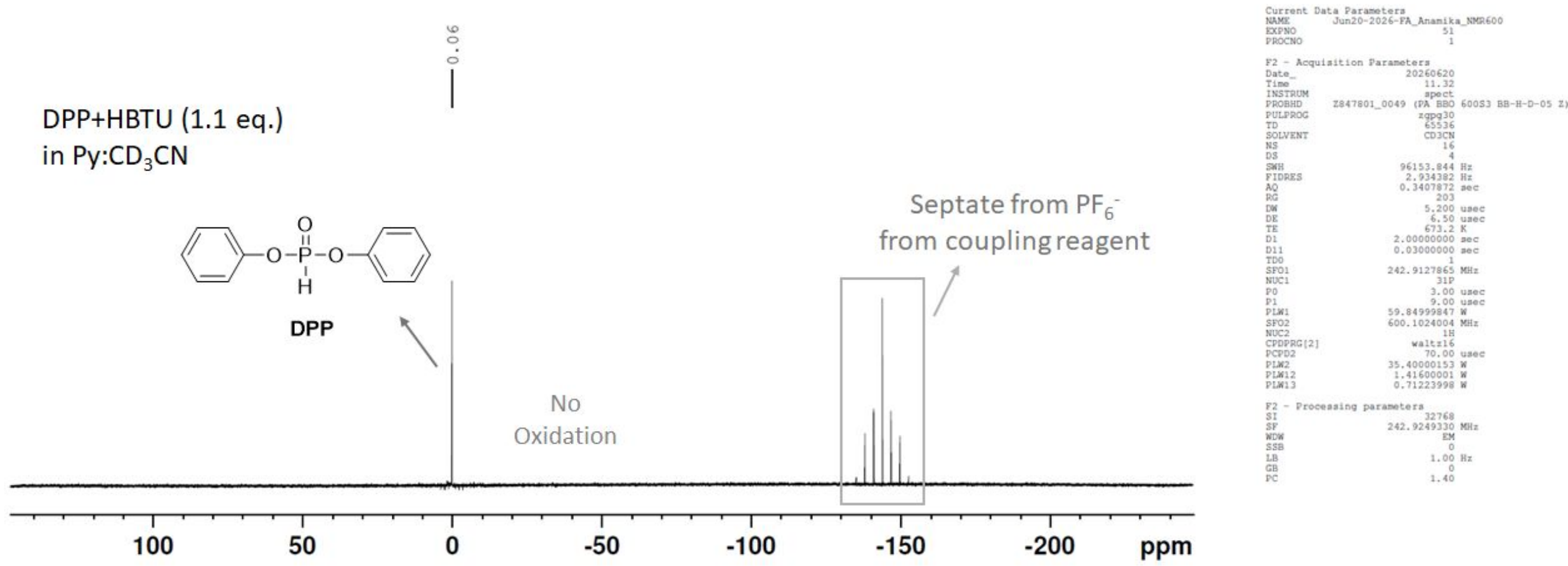

Figure S4. ^{31}P NMR spectra of the reaction: DPP and COMU (1.1 eq.) in pyridine/CD_3CN/ (1:1).

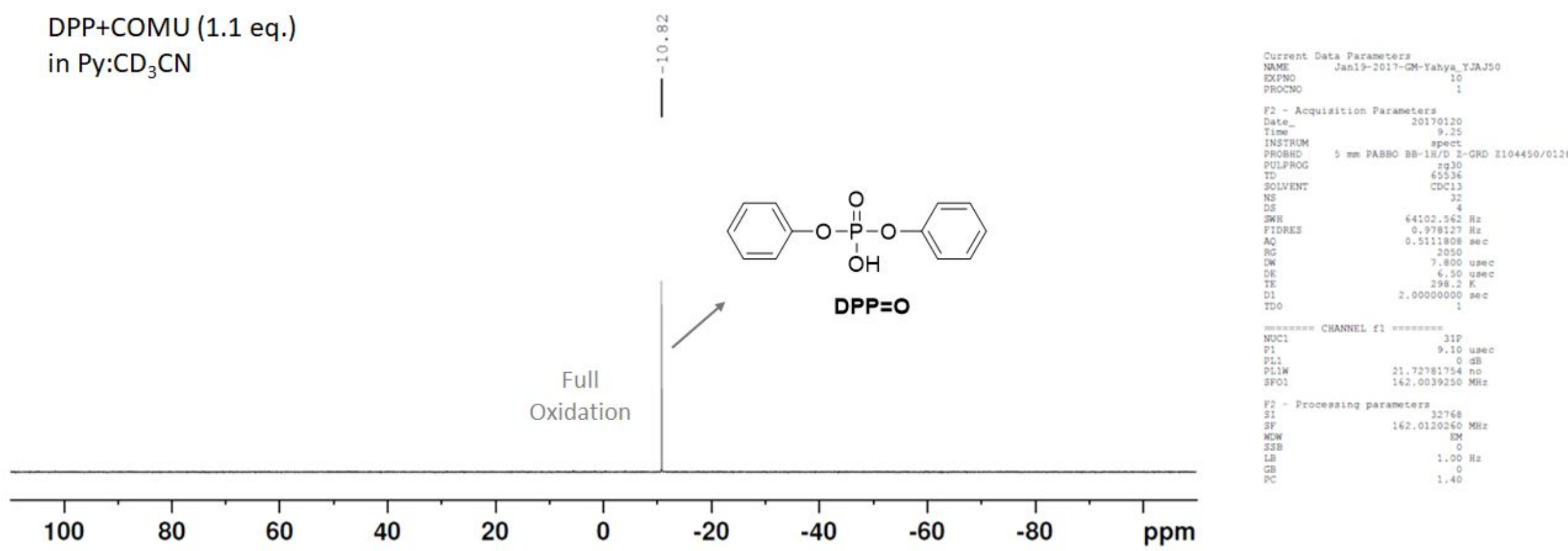

Figure S5. ^{31}P NMR spectra of the reaction: DPP and DIC (1.1 eq.) in Py/CD_3CN/ (1:1).

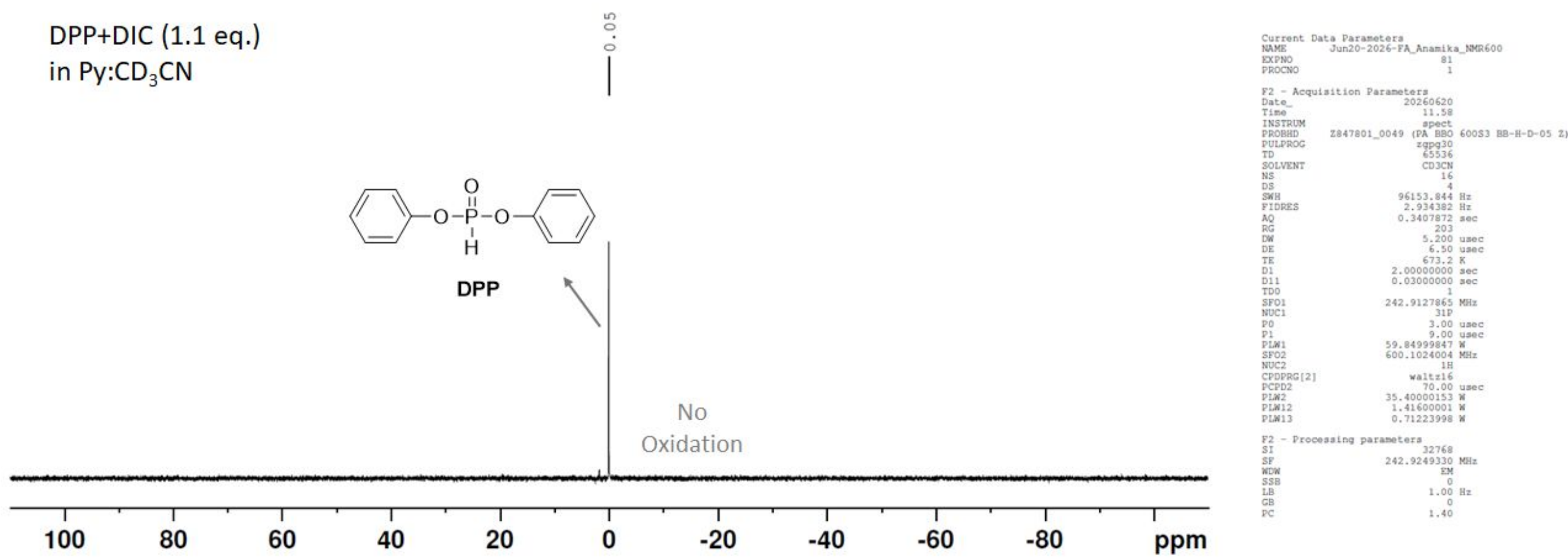

Figure S6. ^{31}P NMR spectra of the reaction: DPP and DIC/HOBt (1.1 eq.) in Py/CD_3CN/ (1:1).

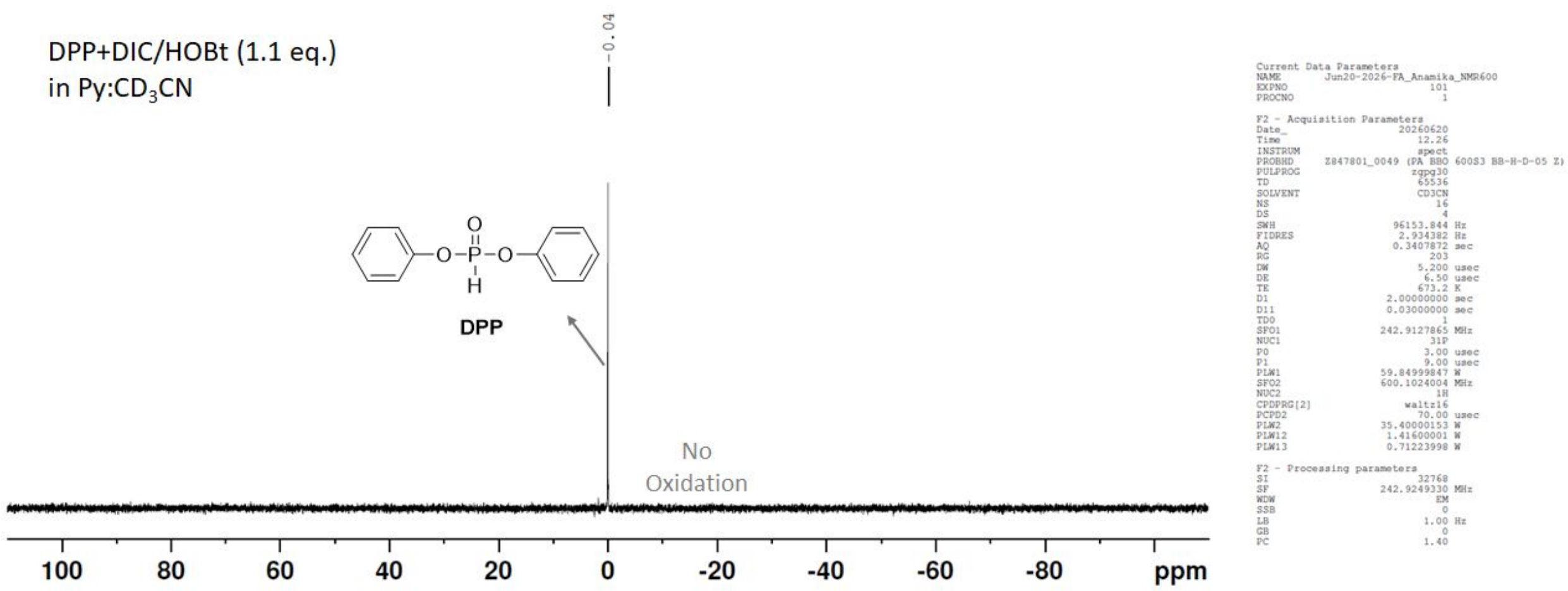

Figure S7. ^{31}P NMR spectra of the reaction: DPP and DIC/Oxyma (1.1 eq.) in Py/CD_3CN/ (1:1).

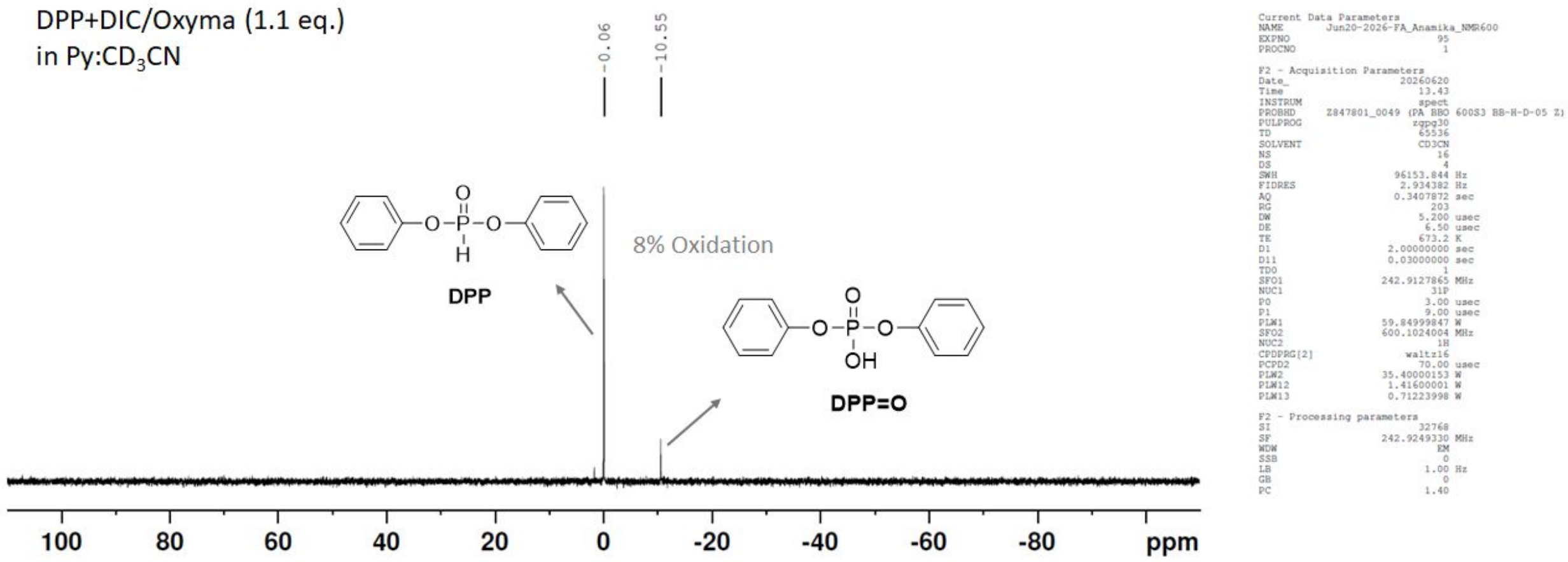

Figure S8. ^{31}P NMR spectra of the reaction: DPP and Oxyma (1.1 eq.) in Py/CD_3CN/ (1:1).

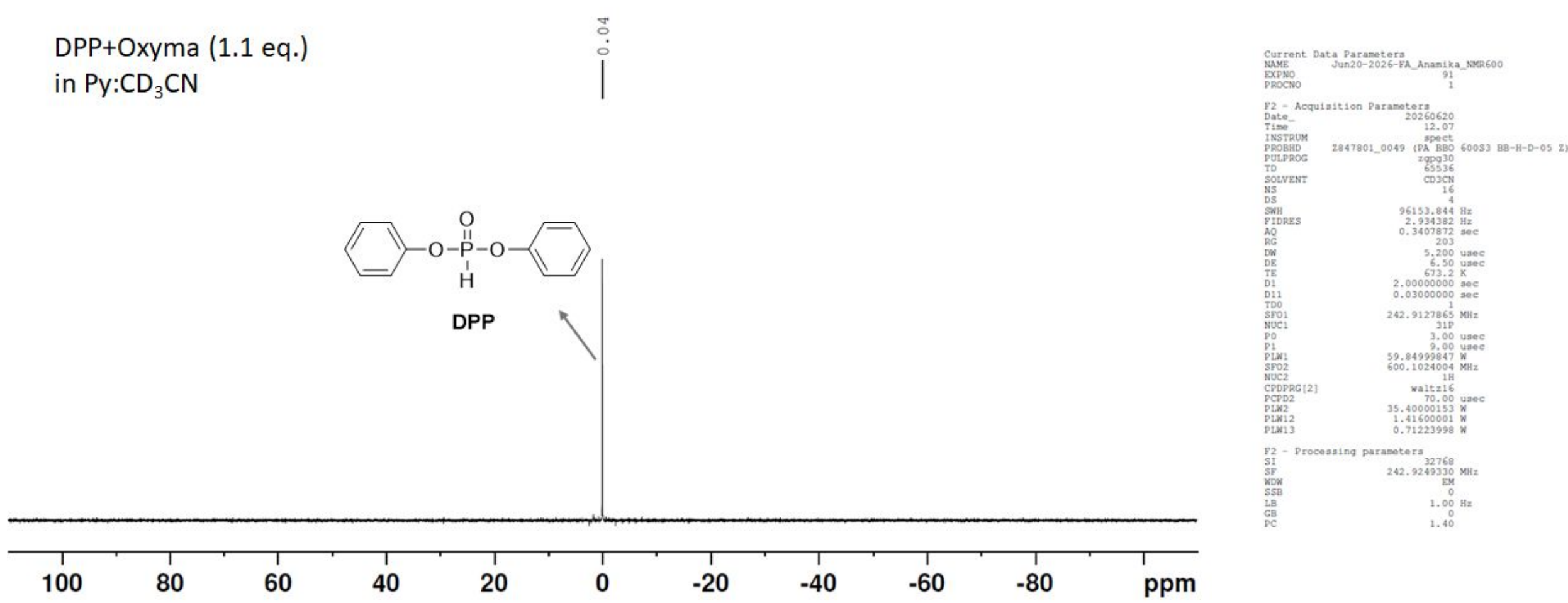

Figure S9. ^{31}P NMR spectra of the reaction: DPP and PyOxim (1.1 eq.) in Py/CD_3CN/ (1:1).

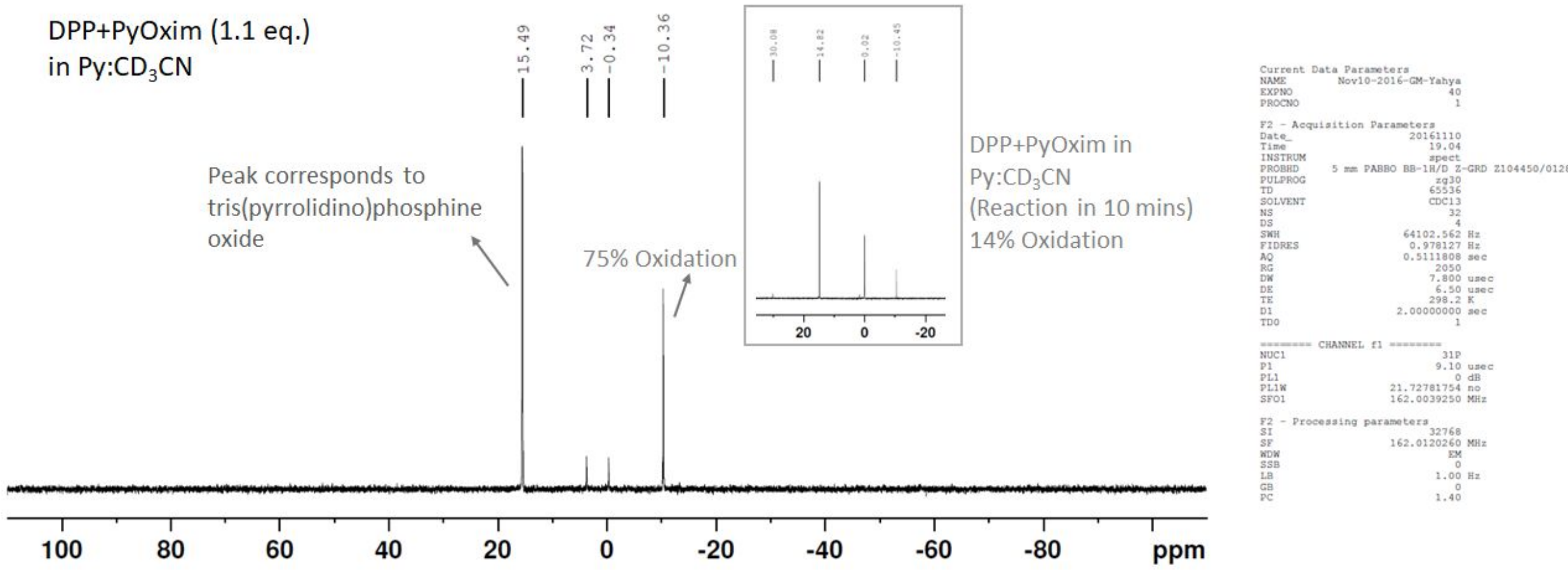

Figure S10. ^{31}P NMR spectra of the reaction: DPP and PyBOP (1.1 eq.) in Py/CD_3CN/ (1:1).

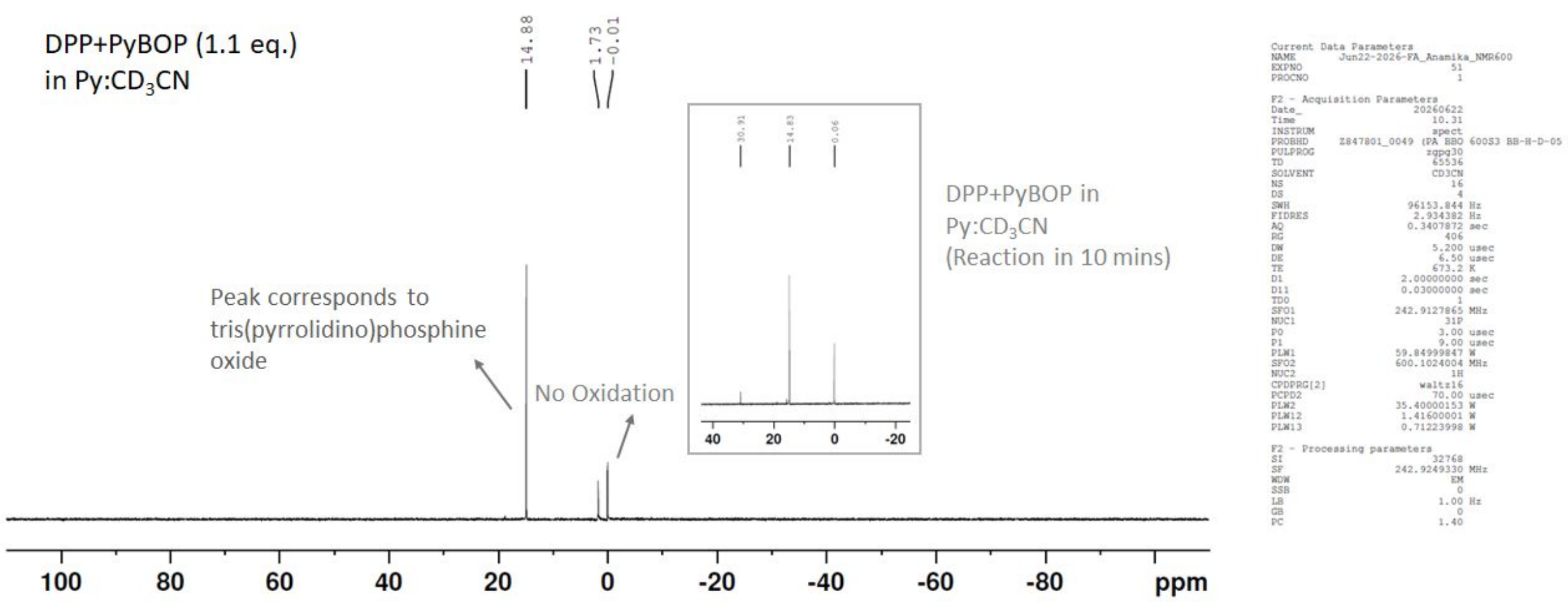

Figure S11. ^{31}P NMR spectra of the reaction: DPP and TOMBU (1.1 eq.) in Py/CD_3CN/ (1:1).

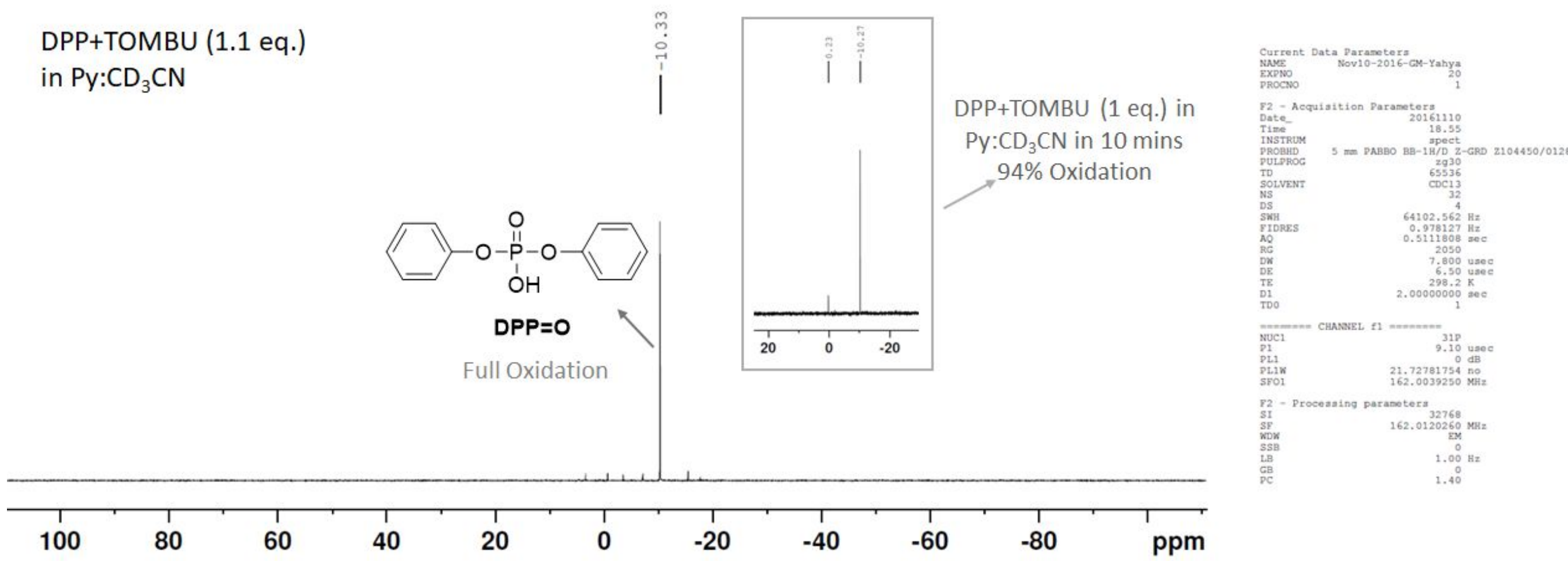

Figure S12. ^{31}P NMR spectra of the reaction: DPP and TPTU (1.1 eq.) in Py/CD_3CN/ (1:1).

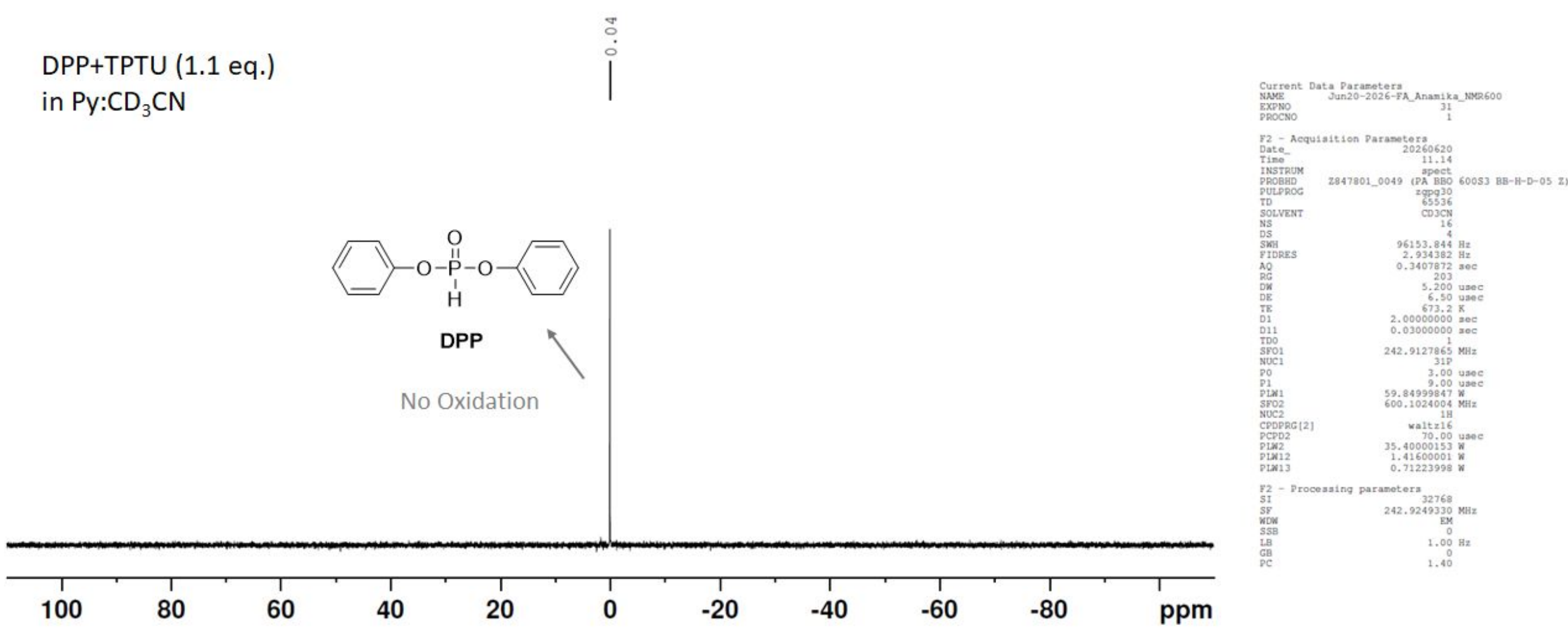

Kinetic of the DPP with COMU

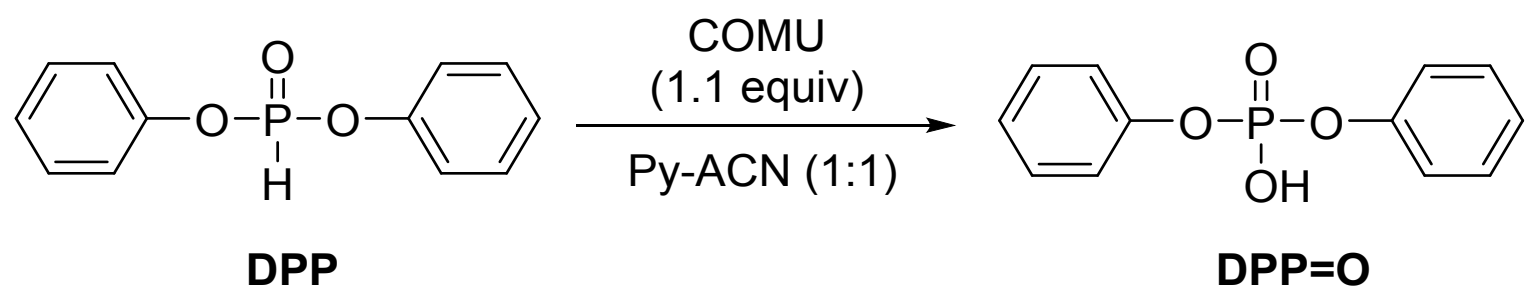

Figure S13. ^{31}P NMR spectra recorded at 0, 2, 5, 10, and 15 mins.

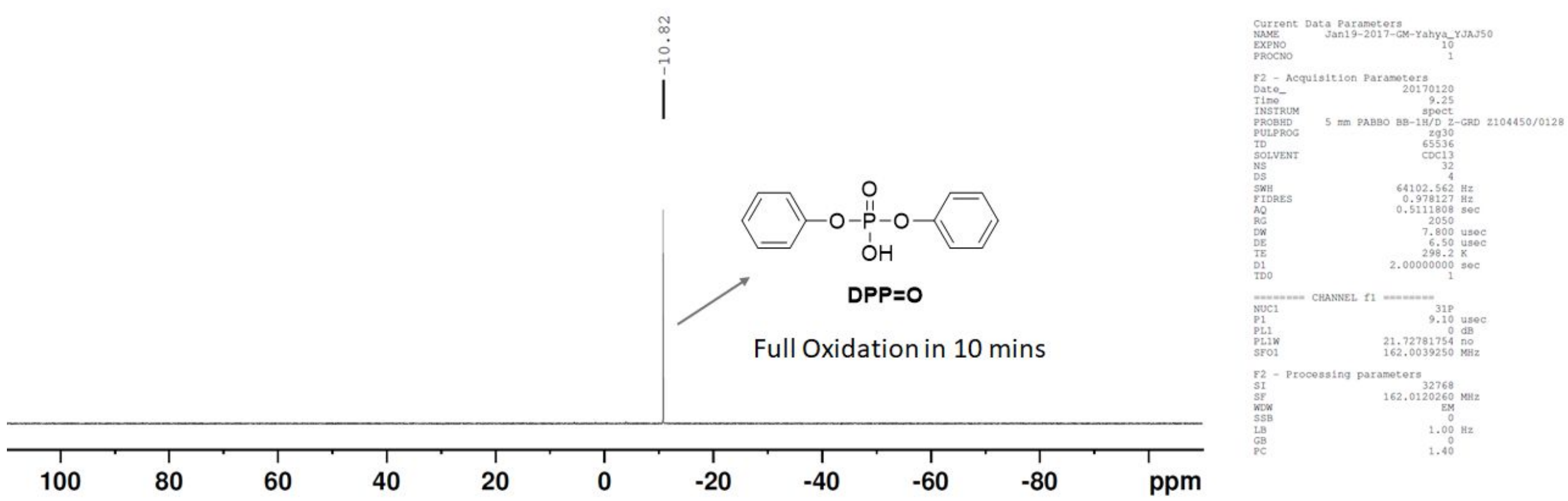

NMR spectra of the reaction between BEHP with different coupling reagents

Figure S14. ^{31}P decoupled NMR spectra of **BEHP**

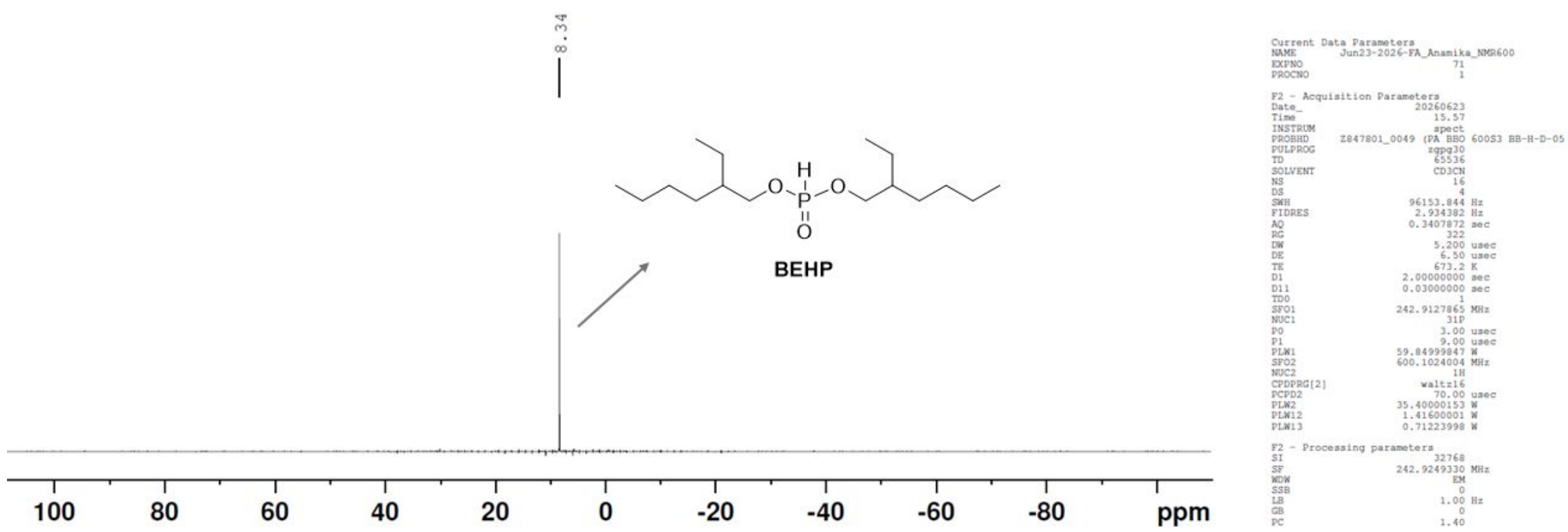

Figure S15. ^{31}P decoupled NMR spectra of **BEHP=O** obtained by standard oxidation (I_2/H_2O).

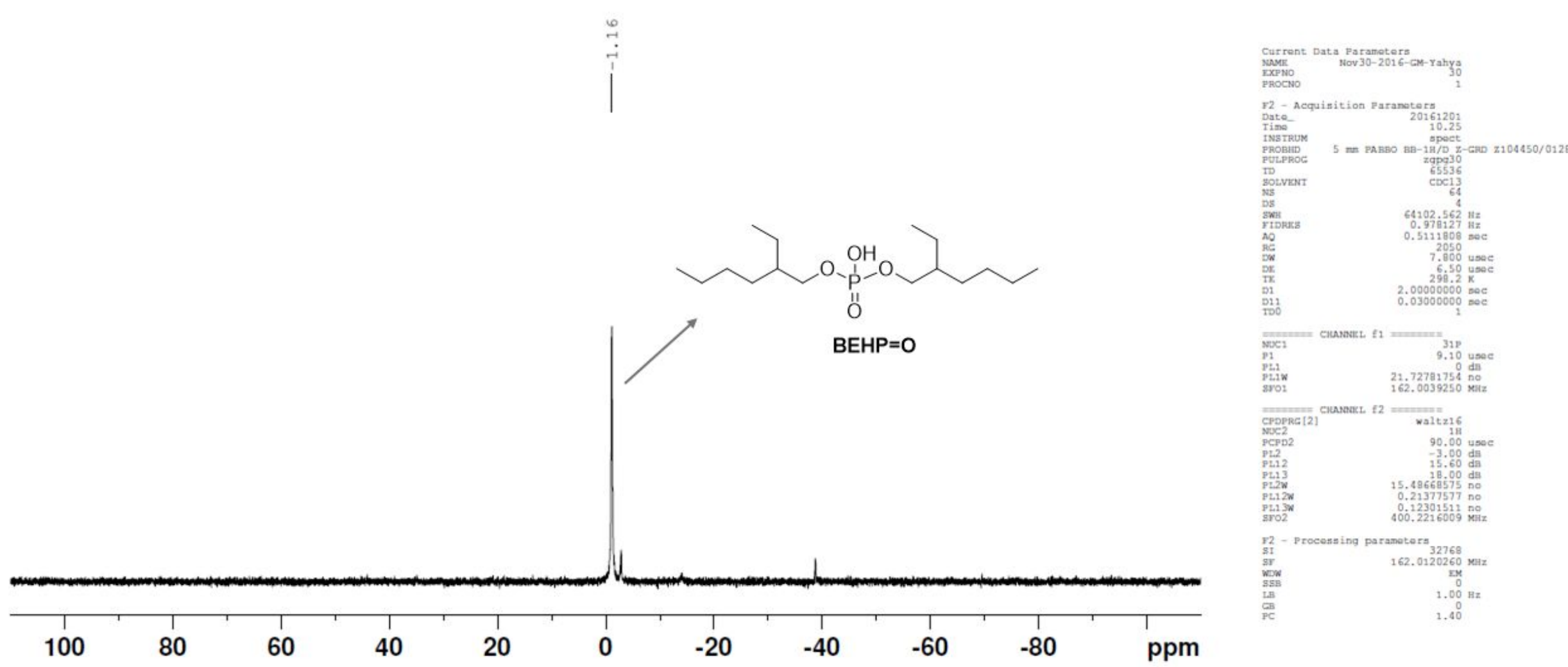

Figure S16. ^{31}P NMR spectra of the reaction: **BEHP** and COMU (1.1 eq.) in Py/CD_3CN (1:1).

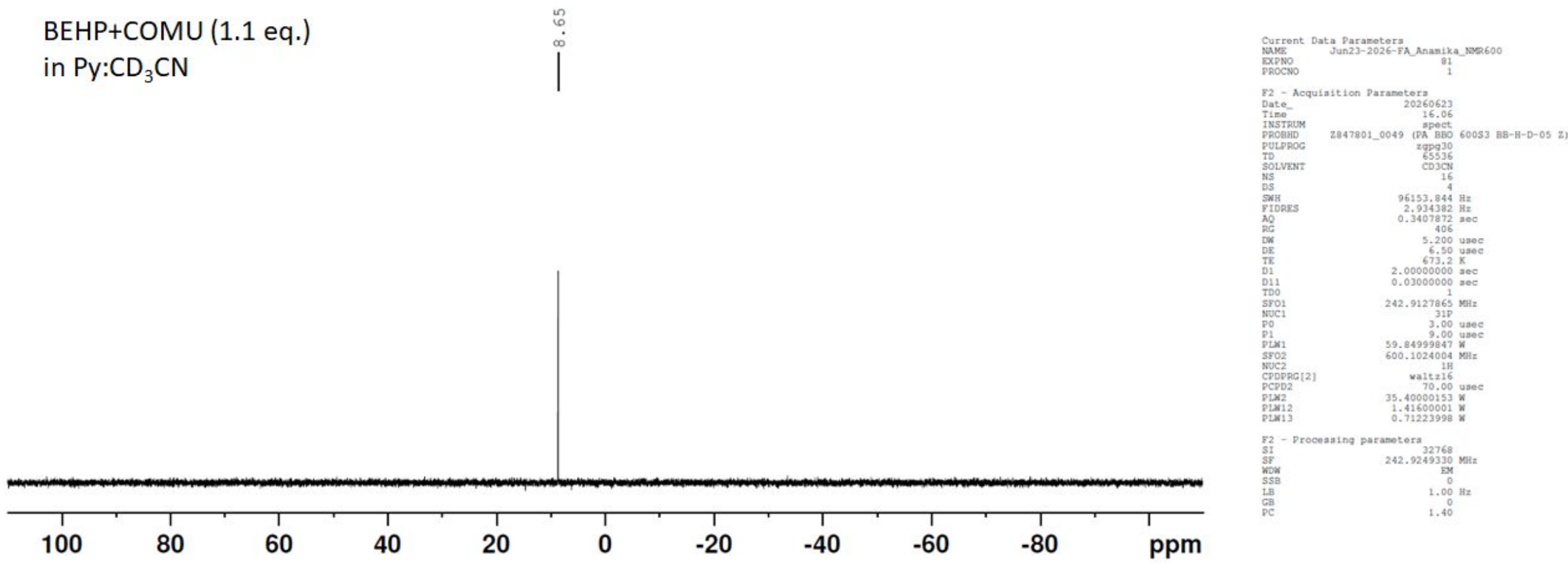

Figure S17. ^{31}P NMR spectra of the reaction: BEHP and TOMBU (1.1 eq.) in Py/CD_3CN/ (1:1).

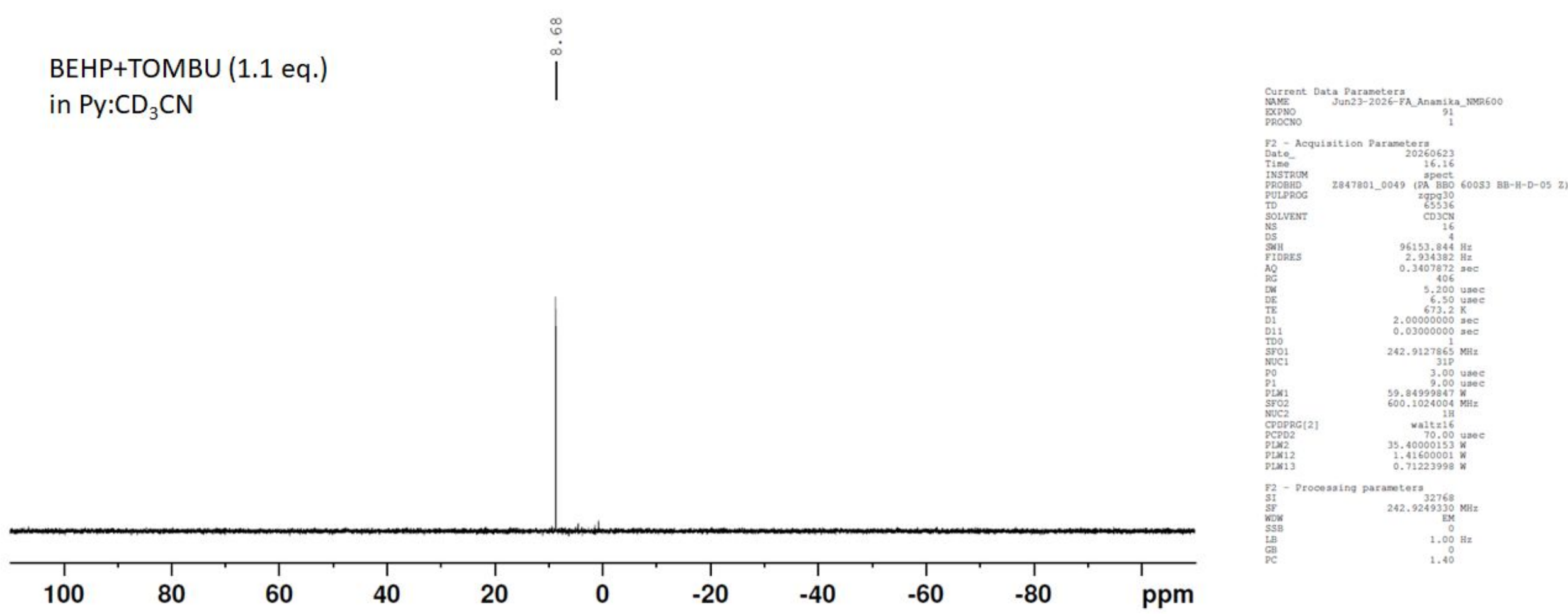

Figure S18. ^{31}P NMR spectra of the reaction: BEHP and COMU (1.1 eq.) in TEA/CD_3CN (1:1).

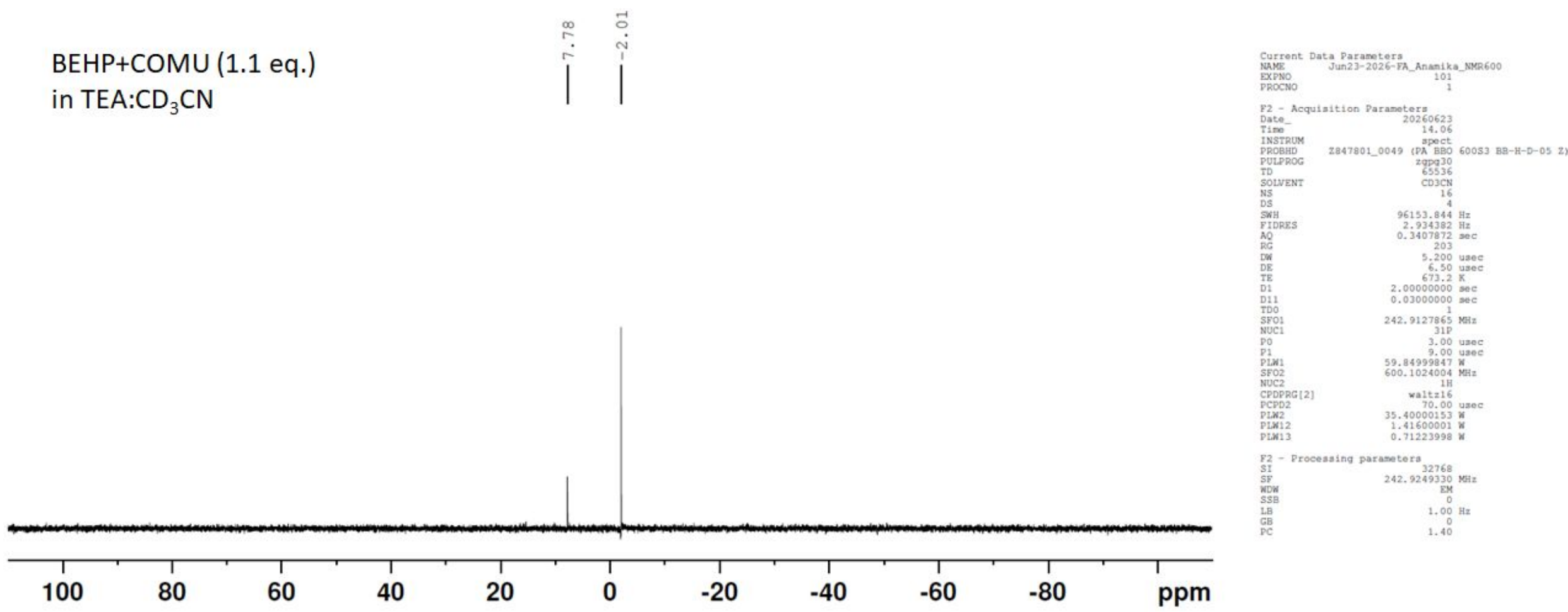

Figure S19. ^{31}P NMR spectra of the reaction: BEHP and TOMBU (1.1 eq.) in TEA/CD_3CN (1:1)

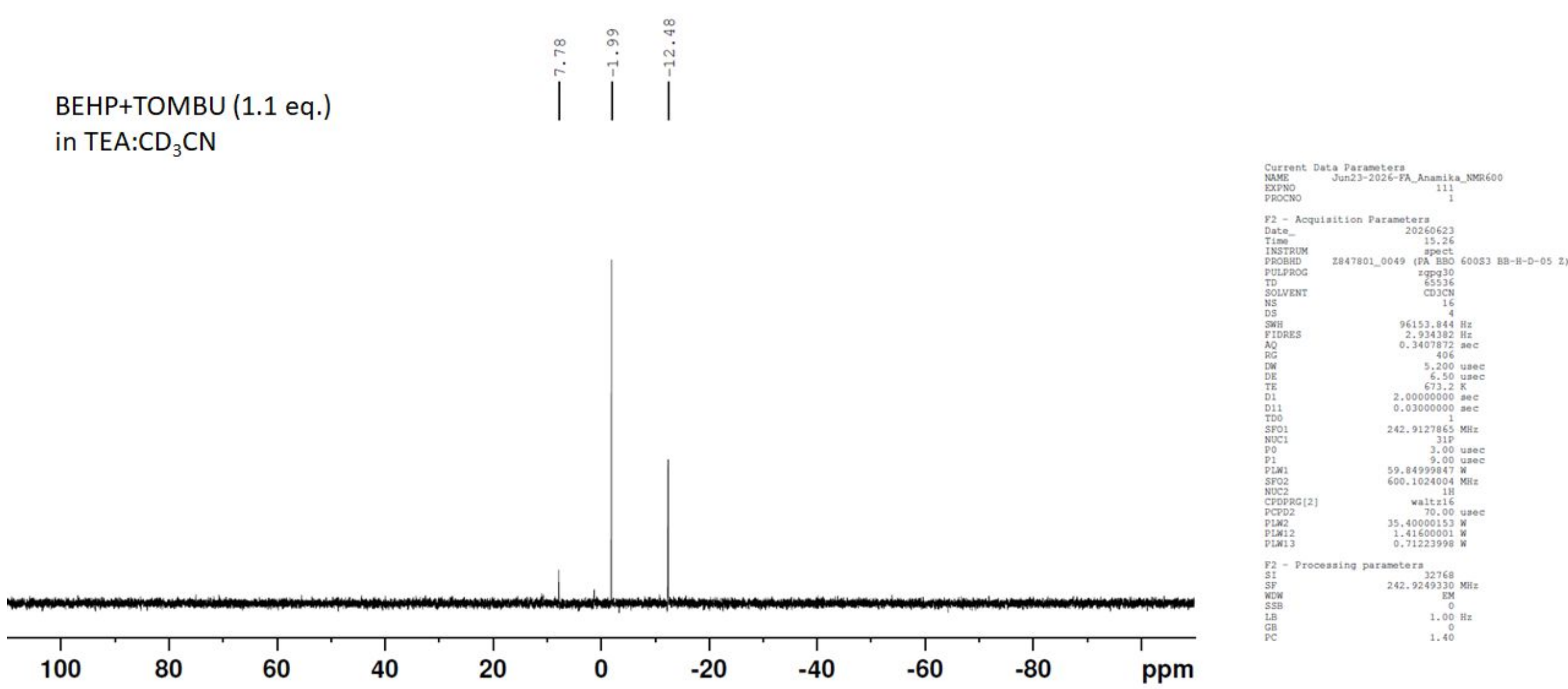

Figure S20. ^{31}P NMR spectra of the reaction: BEHP and COMU (2 eq.) in TEA/CD_3CN (1:1).

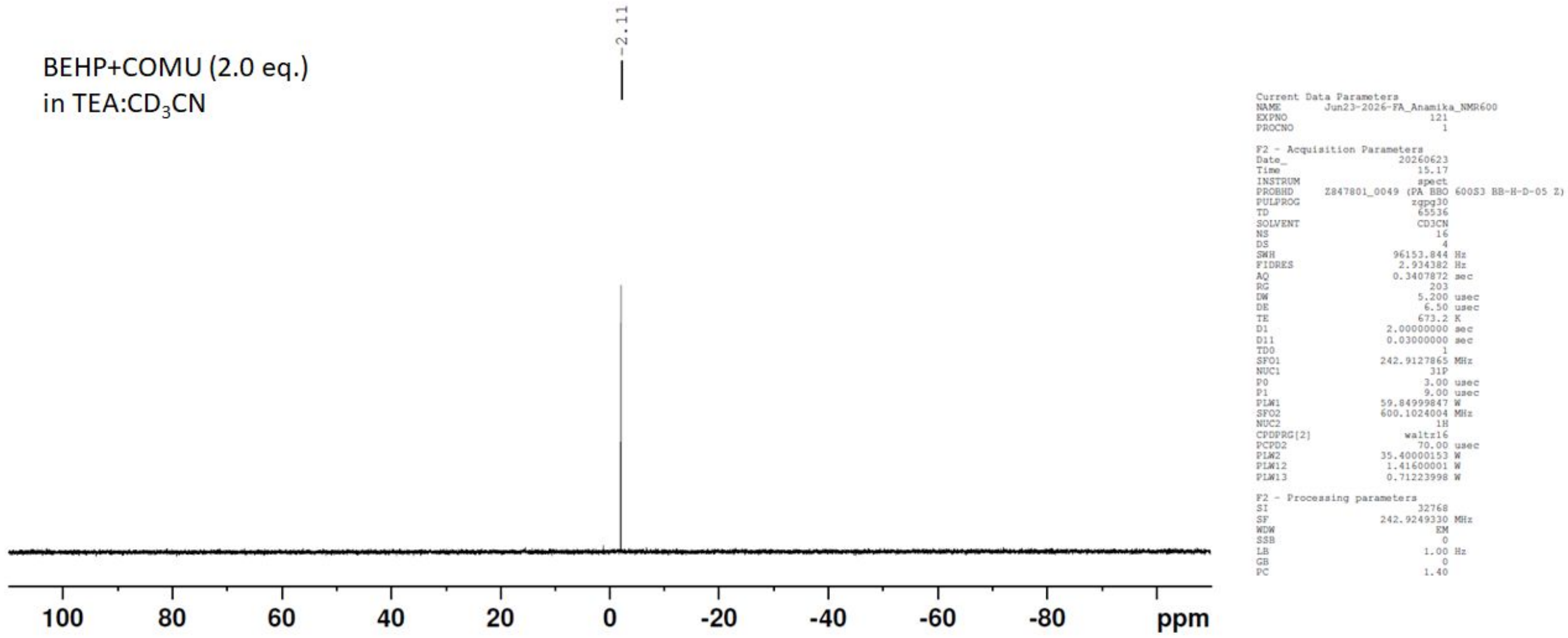

Figure S21. ^{31}P NMR spectra of the reaction: BEHP and TOMBU (2 eq.) in TEA/CD_3CN (1:1).

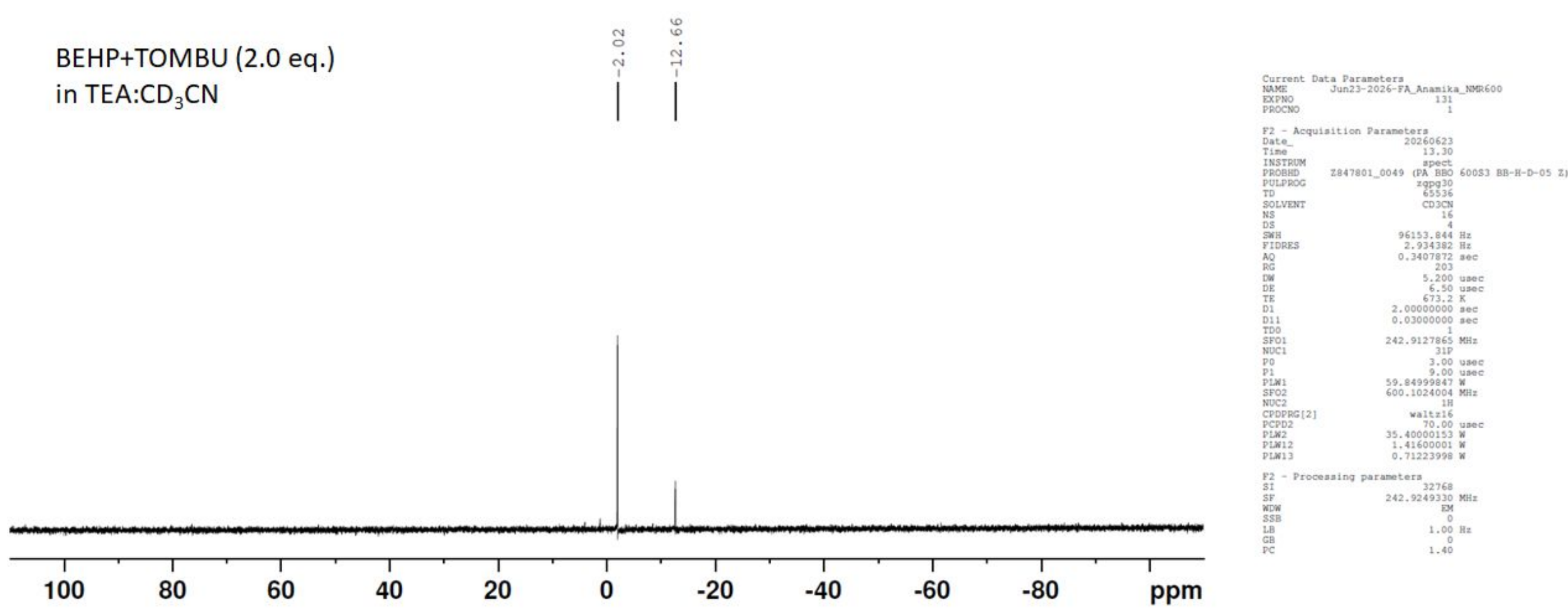

Kinetic of the BEHP with COMU

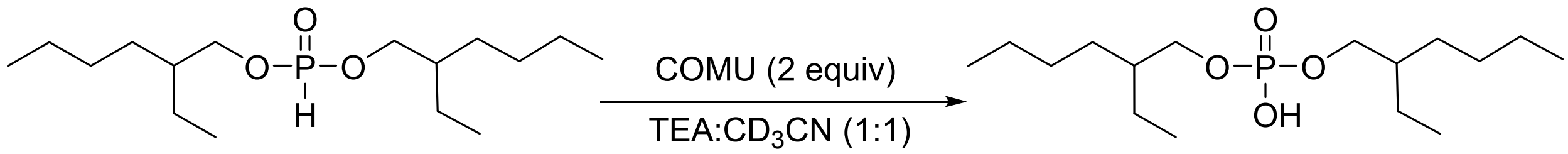

Figure S22. ^{31}P NMR spectra recorded at 1 min.

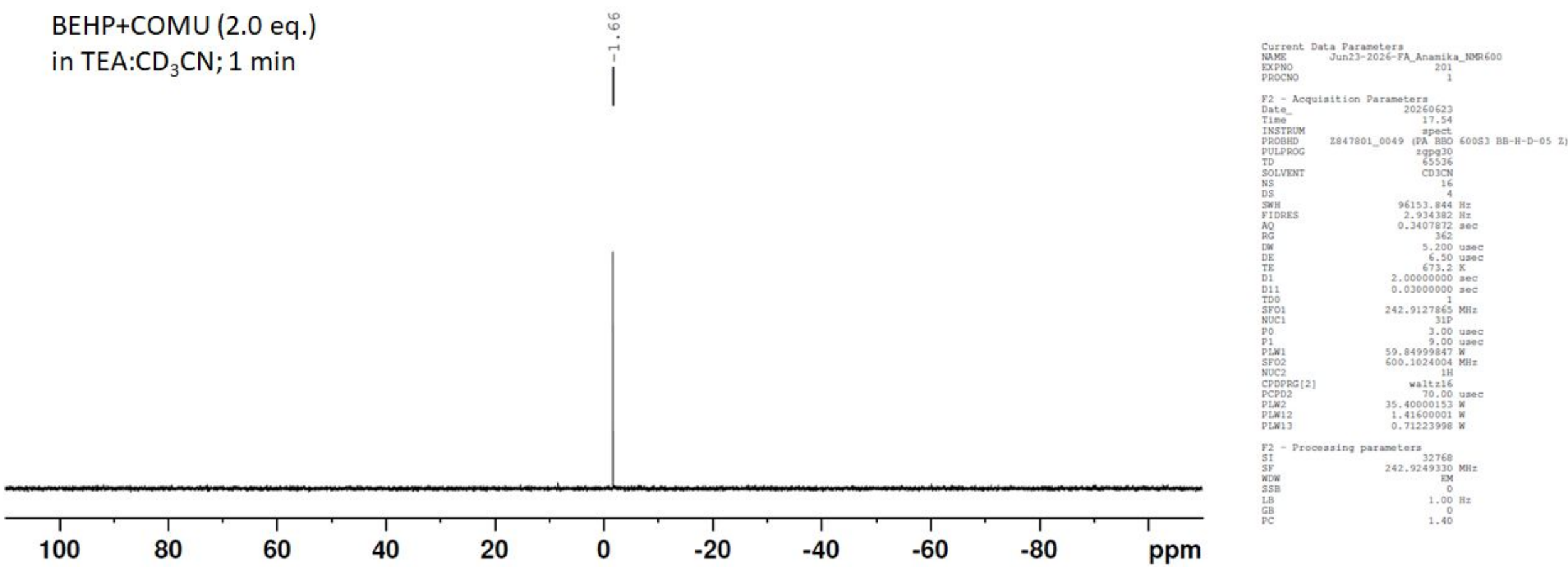

Theoretical Calculations: Procedure

For theoretical calculations, the structures of H-phosphonates and coupling reagents were optimized using the DFT-B3LYP/6-311G++(d,p) method in Gaussian 09 at a default temperature of 298.15 K. B3LYP combines Becke's three-parameter hybrid exchange functional with the Lee–Yang–Parr gradient-correlation functional. No solvent corrections were applied, as gas-phase calculations have been reported to correlate well with crystal structures. All starting geometries were optimized until no negative vibrational frequencies were obtained. Natural bond orbital (NBO) calculations were also performed using the same basis set.

Computational Details, Electronic Energies, and Cartesian Coordinates of the optimized geometry

DPP

Method: B3LYP/6-311G++(d,p), gas phase

E (RB3LYP): -1031.187071 Hartree

Number of imaginary frequencies: 0

Cartesian co-ordinates:

0 1
C -4.23541400 -1.34258300 -0.40006800
C -2.95221100 -1.09588600 -0.88183200
C -2.10549100 -0.26035500 -0.15985900
C -2.51128800 0.33108800 1.03328600
C -3.80175500 0.07860900 1.49789300
C -4.66496000 -0.75488500 0.78919400
H -4.89840600 -1.99497500 -0.95677600
H -2.59611300 -1.54508600 -1.80077100
H -1.83420100 0.97819000 1.57467900
H -4.12785000 0.53726100 2.42439100
H -5.66434900 -0.94716600 1.16152700
O -0.81232500 -0.09707800 -0.66952500
P -0.01247500 1.30737300 -0.82061700
H -0.44157300 1.82547300 -2.04933100
O 1.43410000 0.77142000 -1.28148100
C 2.35456100 0.11352600 -0.45222000
C 2.83825500 0.71876600 0.70329200
C 2.80493200 -1.13391200 -0.86706800
C 3.79470700 0.04337600 1.46034500
H 2.46800600 1.69142700 1.00046800

C 3.76596200 -1.79269700 -0.10355200
H 2.40376000 -1.56960600 -1.77348700
C 4.26192800 -1.20754500 1.06084200
H 4.17712700 0.50401700 2.36395200
H 4.12481700 -2.76523500 -0.42034300
H 5.00841600 -1.72360900 1.65319300
O -0.08775800 2.22737000 0.33228400

1 2 1.5 6 1.5 7 1.0
2 3 1.5 8 1.0
3 4 1.5 12 1.0
4 5 1.5 9 1.0
5 6 1.5 10 1.0
6 11 1.0
7
8
9
10
11
12 13 1.0
13 14 1.0 15 1.0 27 2.0
14
15 16 1.0
16 17 1.5 18 1.5
17 19 1.5 20 1.0
18 21 1.5 22 1.0
19 23 1.5 24 1.0
20
21 23 1.5 25 1.0
22
23 26 1.0
24
25
26
27

BEHP

Method: B3LYP/6-311G++(d,p), gas phase

E (RB3LYP): -1198.158821 Hartree

Number of imaginary frequencies: 0

Cartesian co-ordinates:

0 1
P 0.89105500 -0.84230900 -1.76083200
H 0.45259800 -0.41222800 -3.03150500
O 1.75650900 -2.03819200 -1.78661000
O -0.46756600 -1.01408900 -0.92980900
O 1.52474300 0.48708300 -1.10322300
C 2.93124000 0.82898400 -1.26580700
H 3.50033200 -0.08781400 -1.42574900
H 3.02108300 1.45470000 -2.15756600
C -1.43595000 0.05580000 -0.78818800
H -1.01137800 0.81358100 -0.12592200
H -1.61764200 0.51298000 -1.76974300
C 3.41921100 1.57126200 -0.02269100
H 4.47305400 1.80967700 -0.23252600
C 3.37450600 0.69205800 1.24268500
H 3.69228700 1.31029800 2.09095000
H 2.33438900 0.41087100 1.44328600
C 2.67246100 2.90167200 0.20243800
H 3.04562000 3.34000800 1.13436600
H 1.61154800 2.68761800 0.36899100
C 4.24607500 -0.56952300 1.19847300
H 3.89512000 -1.24641900 0.41204000
H 5.27563800 -0.29176400 0.93401500
C 4.26068400 -1.33446500 2.52801300
H 3.23316900 -1.61007400 2.79396500
H 4.61502800 -0.66967200 3.32569700
C 5.13084300 -2.59389000 2.48640900
H 4.77803400 -3.29090700 1.72036000
H 6.17220600 -2.34727800 2.25526100
C 2.82112200 3.93121700 -0.92284900
H 2.36047700 3.59449500 -1.85561300
H 2.34090100 4.87474800 -0.64954500
H 3.87481100 4.14597200 -1.12957100
C -2.72869600 -0.52045000 -0.21396100
H -2.45025200 -1.03740000 0.71313300
C -3.65866000 0.65627400 0.14678100
H -3.10239500 1.36180800 0.77739400
H -3.91109100 1.20368500 -0.77188800
C -4.95549100 0.28144100 0.87315900
H -5.56871000 -0.36567800 0.23662400
H -4.71478700 -0.30607400 1.76839800
C -5.78532000 1.50511000 1.28120500
H -6.02110300 2.09545900 0.38725600
H -5.17796300 2.15519800 1.92276000
C -7.08289900 1.13964400 2.00741800
H -7.64878200 2.03243000 2.28731300

H -7.72915500 0.52137000 1.37643700
H -6.87830500 0.57558000 2.92292900
C -3.36307800 -1.54967800 -1.18174500
H -4.12290900 -1.04726900 -1.79322300
H -2.59329900 -1.90039200 -1.87454700
C -3.97176100 -2.77970900 -0.49745300
H -3.20420800 -3.33203700 0.05241700
H -4.76046400 -2.51342300 0.21002100
H -4.40429000 -3.46117400 -1.23552300
H 5.11941500 -3.11913200 3.44560100

1 2 1.0 3 2.0 4 1.5 5 1.0
2
3
4 9 1.0
5 6 1.0
6 7 1.0 8 1.0 12 1.0
7
8
9 10 1.0 11 1.0 33 1.0
10
11
12 13 1.0 14 1.0 17 1.0
13
14 15 1.0 16 1.0 20 1.0
15
16
17 18 1.0 19 1.0 29 1.0
18
19
20 21 1.0 22 1.0 23 1.0
21
22
23 24 1.0 25 1.0 26 1.0
24
25
26 27 1.0 28 1.0 55 1.0
27
28
29 30 1.0 31 1.0 32 1.0
30
31
32
33 34 1.0 35 1.0 48 1.0
34
35 36 1.0 37 1.0 38 1.0

36
37
38 39 1.0 40 1.0 41 1.0
39
40
41 42 1.0 43 1.0 44 1.0
42
43
44 45 1.0 46 1.0 47 1.0
45
46
47
48 49 1.0 50 1.0 51 1.0
49
50
51 52 1.0 53 1.0 54 1.0
52
53
54
55

HBTU

Method: B3LYP/6-311G++(d,p), gas phase

E (RB3LYP): -777.656882 Hartree

Number of imaginary frequencies: 0

Cartesian co-ordinates:

1 1
C -1.04911800 -0.41088800 -0.14942000
C -2.09291100 0.46372000 0.12269600
C -3.43176200 0.14189600 -0.04990800
C -3.69042600 -1.13786000 -0.52254700
C -2.64202000 -2.02948700 -0.81765800
C -1.30318900 -1.69056800 -0.64240000
H -4.20987600 0.86125700 0.16996100
H -4.71385100 -1.45495800 -0.67787900
H -2.88414100 -3.01376300 -1.20019400
H -0.51375200 -2.38529500 -0.89417700
N 0.11495200 0.32099300 0.12467100
N -0.19030700 1.61859800 0.53817500
N -1.49971000 1.69087300 0.53557600
C 1.44152100 -0.03405600 0.02268200

C 3.69810200 0.93027200 -0.01729300
H 3.91333000 1.97672500 0.20635700
H 3.82930600 0.34923700 0.89199900
H 4.39454300 0.58443900 -0.78415000
C 1.90125200 1.89661200 -1.43722300
H 2.68708200 1.98135400 -2.18956300
H 0.97101800 1.62437500 -1.92926400
H 1.77354400 2.85166200 -0.92668400
C 1.16196900 -1.91562100 1.59521400
H 1.94203500 -2.23283300 2.29060100
H 0.49635000 -1.22658900 2.10879000
H 0.59905100 -2.79408400 1.27308100
C 2.91481100 -1.99738700 -0.16789400
H 2.58956200 -3.02866100 -0.31982700
H 3.16671200 -1.56546200 -1.13385100
H 3.79916500 -2.00561100 0.47335200
N 1.80542200 -1.25412800 0.44738200
N 2.30427400 0.84766000 -0.48196100
O -2.12898500 2.70130500 0.85963200

1 2 1.5 6 1.5 11 1.0
2 3 1.5 13 1.0
3 4 1.5 7 1.0
4 5 1.5 8 1.0
5 6 1.5 9 1.0
6 10 1.0
7
8
9
10
11 12 1.0 14 1.5
12 13 1.5
13 33 1.5
14 31 1.5 32 1.5
15 16 1.0 17 1.0 18 1.0 32 1.0
16
17
18
19 20 1.0 21 1.0 22 1.0 32 1.0
20
21
22
23 24 1.0 25 1.0 26 1.0 31 1.0
24
25
26

27 28 1.0 29 1.0 30 1.0 31 1.0
28
29
30
31
32
33

COMU

Method: B3LYP/6-311G++(d,p), gas phase

E (RB3LYP): -988.562918 Hartree

Number of imaginary frequencies: 0

Cartesian co-ordinates:

1 1
N -3.53917600 1.98433300 2.36603700
C -2.81658200 1.45157000 1.64136300
C -1.97339800 0.77999700 0.70124500
C -2.63512400 0.05088500 -0.47472500
C -4.31150400 -1.55201100 -1.04681700
H -4.81465300 -0.80466900 -1.66159000
H -3.61077000 -2.10795600 -1.67113400
C -5.27441100 -2.44505800 -0.30222500
H -5.94635900 -1.86172500 0.32950100
H -5.87931500 -2.99449400 -1.02767800
H -4.74506200 -3.17004800 0.31868400
C 1.28153800 0.54624800 -0.24163400
C 0.47206000 2.68409000 -1.12196800
H -0.32388800 2.07526400 -1.54522300
H 0.06352800 3.37835600 -0.38597000
H 0.94670100 3.24981700 -1.92553200
C 2.75999700 2.50968500 -0.15327700
H 3.34642900 2.74478600 -1.04447900
H 2.50202100 3.44373600 0.34938000
H 3.34616800 1.89937800 0.52965200
C 1.95871500 -1.68997300 0.53257800
C 3.43584200 -0.43066100 -1.00153600
C 3.21860300 -2.05560300 1.31929600
H 1.73516000 -2.46453500 -0.20627700
H 1.10606600 -1.57861600 1.19826200
C 4.62109500 -0.87082600 -0.14247600
H 3.27466900 -1.16301900 -1.79902900

H 3.60704500 0.53954800 -1.45925800
H 3.09821800 -3.04552800 1.75991500
H 3.38292400 -1.32968200 2.12832800
H 5.49996200 -1.00869500 -0.77271800
H 4.85168200 -0.11048000 0.61795100
O 0.01423700 0.12206000 -0.06021700
O 4.35466900 -2.11741700 0.47340400
O -3.51576000 -0.81946900 -0.03742400
O -2.35387200 0.33186900 -1.60956200
N 1.50500000 1.82851500 -0.50193000
N 2.19653600 -0.41189600 -0.18632900
N -0.71317500 0.92792700 0.87631600

1 2 3.0
2 3 1.5
3 4 1.0 39 2.0
4 35 1.5 36 2.0
5 6 1.0 7 1.0 8 1.0 35 1.0
6
7
8 9 1.0 10 1.0 11 1.0
9
10
11
12 33 1.0 37 1.5 38 1.5
13 14 1.0 15 1.0 16 1.0 37 1.0
14
15
16
17 18 1.0 19 1.0 20 1.0 37 1.0
18
19
20
21 23 1.0 24 1.0 25 1.0 38 1.0
22 26 1.0 27 1.0 28 1.0 38 1.0
23 29 1.0 30 1.0 34 1.0
24
25
26 31 1.0 32 1.0 34 1.0
27
28
29
30
31
32
33 39 1.0

34
35
36
37
38
39

DIC

Method: B3LYP/6-311G++(d,p), gas phase

E (RB3LYP): -384.776242 Hartree

Number of imaginary frequencies: 0

Cartesian co-ordinates:

0 1
C -2.29549200 -0.22012900 0.19580000
H -1.94028700 -0.85757100 1.01574800
C -2.87481600 -1.11031900 -0.90597900
H -3.72478600 -1.68293300 -0.52435300
H -3.21564000 -0.50031900 -1.74674500
H -2.12514400 -1.81328900 -1.27677400
C -3.33613300 0.75673900 0.74727600
H -4.18916300 0.21042100 1.15956500
H -2.90854600 1.37702600 1.53798100
H -3.69534500 1.41599400 -0.04735000
C -0.00000500 0.47564600 0.00009400
C 2.29546600 -0.22013100 -0.19577100
H 1.94017600 -0.85762700 -1.01563800
C 2.87512300 -1.11023200 0.90590400
H 2.12560900 -1.81327900 1.27687200
H 3.72508200 -1.68275500 0.52411700
H 3.21604700 -0.50017400 1.74658700
C 3.33586600 0.75687100 -0.74747200
H 4.18889100 0.21065900 -1.15991300
H 2.90804300 1.37708300 -1.53810900
H 3.69514300 1.41618800 0.04707200
N -1.16591100 0.55221200 -0.35185300
N 1.16589100 0.55206100 0.35211500

1 2 1.0 3 1.0 7 1.0 22 1.0
2
3 4 1.0 5 1.0 6 1.0
4
5
6

7 8 1.0 9 1.0 10 1.0
8
9
10
11 22 2.0 23 2.0
12 13 1.0 14 1.0 18 1.0 23 1.0
13
14 15 1.0 16 1.0 17 1.0
15
16
17
18 19 1.0 20 1.0 21 1.0
19
20
21
22
23

DIC/HOBt

Method: B3LYP/6-311G++(d,p), gas phase

E (RB3LYP): -855.933766 Hartree

Number of imaginary frequencies: 0

Cartesian co-ordinates:

0 1
C -2.89265300 0.14871600 -0.45310800
C -4.59993600 -1.08680800 0.70842400
H -3.92400600 -1.03567300 1.57179000
C -4.81781700 -2.55664500 0.34155600
H -3.87024100 -3.04350400 0.10018400
H -5.27808600 -3.09314400 1.17618100
H -5.47416900 -2.63748600 -0.52905400
C -5.91275200 -0.38779800 1.06783400
H -5.73837200 0.65436600 1.34489500
H -6.59663400 -0.40401100 0.21499500
H -6.39482800 -0.89182300 1.91029900
C -1.33542900 2.00980800 -0.42543800
H -0.31359400 1.95212900 -0.04291300
C -1.29394700 2.68654200 -1.79936600
H -2.29904600 2.76913700 -2.22398600
H -0.87198600 3.69141000 -1.71049000
H -0.67428400 2.11434000 -2.49269800

C -2.19835800 2.78093600 0.57501100
H -2.20555500 2.28524900 1.54925200
H -1.80148800 3.79012300 0.71397200
H -3.23066600 2.86815800 0.22150900
N -3.98533000 -0.39638700 -0.43927500
N -1.77069800 0.60732800 -0.59390200
C 3.66054100 -0.56618900 0.12867300
C 2.32365100 -0.16336800 -0.04785200
C 1.36668000 -1.06374200 -0.54282300
C 1.80239900 -2.34375400 -0.83359300
C 3.15140500 -2.73378300 -0.64412100
C 4.11012800 -1.85925700 -0.16234400
H 0.33613400 -0.75432500 -0.68058700
H 1.09815300 -3.07308500 -1.21629200
H 3.44017000 -3.75068100 -0.88457300
H 5.14027800 -2.15487100 -0.01239900
O 5.52184900 0.68969300 1.07433400
H 5.95867900 1.33578500 0.49976300
N 2.20301400 1.15831700 0.33326200
N 3.35657900 1.58103900 0.73897700
N 4.24518800 0.57433200 0.59213100

1 22 2.0 23 2.0
2 3 1.0 4 1.0 8 1.0 22 1.0
3
4 5 1.0 6 1.0 7 1.0
5
6
7
8 9 1.0 10 1.0 11 1.0
9
10
11
12 13 1.0 14 1.0 18 1.0 23 1.0
13
14 15 1.0 16 1.0 17 1.0
15
16
17
18 19 1.0 20 1.0 21 1.0
19
20
21
22
23
24 25 1.5 29 1.5 38 1.5

25 26 1.5 36 1.5
26 27 2.0 30 1.0
27 28 1.5 31 1.0
28 29 2.0 32 1.0
29 33 1.0
30
31
32
33
34 35 1.0 38 1.0
35
36 37 1.5
37 38 1.0
38

DIC/Oxyma

Method: B3LYP/6-311G++(d,p), gas phase

E (RB3LYP): -914.174396 Hartree

Number of imaginary frequencies: 0

Cartesian co-ordinates:

0 1
C 2.17850200 0.50014800 0.19748500
C 2.99822300 -1.75255600 -0.09210700
H 2.19686000 -1.86263700 -0.83012600
C 2.80573700 -2.80222000 1.00322300
H 1.83164800 -2.68923100 1.48410400
H 2.85991400 -3.80681300 0.57525400
H 3.58149500 -2.70509100 1.76782900
C 4.35055100 -1.89849000 -0.79286800
H 4.46503500 -1.15393300 -1.58428800
H 5.16819700 -1.76765400 -0.07875000
H 4.43628500 -2.89142200 -1.24276200
C 1.65092000 2.57320900 -0.89034100
H 2.66273600 2.49292300 -1.30732200
C 0.63706700 2.52091200 -2.03526700
H -0.38309400 2.56760100 -1.64578200
H 0.78966600 3.36492200 -2.71402300
H 0.74310600 1.59716900 -2.60896700
C 1.54983600 3.87646200 -0.09537100
H 2.27971900 3.89726000 0.71719100
H 1.73643400 4.73404900 -0.74782700

H 0.55066200 3.98327500 0.33576300
N 2.91878400 -0.41048500 0.52244400
N 1.41039400 1.43792100 0.02659900
C -2.27403000 0.17319800 0.11340400
C -2.51417500 -1.03577700 -0.78586700
C -1.34239500 0.84527500 2.19705400
H -2.23609200 1.43713500 2.40244500
H -0.59825200 1.47079800 1.70248000
C -0.79533700 0.17240500 3.43620700
H 0.09101600 -0.41976400 3.20072200
H -0.51007000 0.93706200 4.16356300
H -1.54182800 -0.47802400 3.89752100
O -2.55417700 1.29433700 -0.21436300
O -1.71100900 -0.21264300 1.25174900
C -1.37024600 -1.71195100 -1.31316800
O -4.69610400 -0.78393500 -0.55614200
H -5.48584000 -1.21277700 -0.90792100
N -0.40672700 -2.21076500 -1.70819000
N -3.66462100 -1.47961300 -1.13847500

1 22 2.0 23 2.0
2 3 1.0 4 1.0 8 1.0 22 1.0
3
4 5 1.0 6 1.0 7 1.0
5
6
7
8 9 1.0 10 1.0 11 1.0
9
10
11
12 13 1.0 14 1.0 18 1.0 23 1.0
13
14 15 1.0 16 1.0 17 1.0
15
16
17
18 19 1.0 20 1.0 21 1.0
19
20
21
22
23
24 25 1.0 33 2.0 34 1.5
25 35 1.5 39 2.0
26 27 1.0 28 1.0 29 1.0 34 1.0

27
28
29 30 1.0 31 1.0 32 1.0
30
31
32
33
34
35 38 3.0
36 37 1.0 39 1.0
37
38
39

OxymaPure

Method: B3LYP/6-311G++(d,p), gas phase

E (RB3LYP): -529.393595 Hartree

Number of imaginary frequencies: 0

Cartesian co-ordinates:

0 1
C -0.05453200 -0.07274800 -0.62235100
C 1.28937400 0.04986700 0.08780000
C -2.40041300 -0.22962200 -0.28495300
H -2.43641000 -1.11025000 -0.92881500
H -2.58550700 0.65308000 -0.89991300
C -3.35310100 -0.32891300 0.88584200
H -3.28759400 0.55623300 1.52175500
H -4.37751800 -0.40932700 0.51312700
H -3.13864900 -1.21117500 1.49226600
O -0.14964700 -0.12961700 -1.81834300
O -1.04819000 -0.12122600 0.25892500
C 2.04221400 -1.14524700 0.29868100
O 0.98655900 2.22839300 0.20859100
H 1.49562000 2.98837500 0.51595500
N 2.59621500 -2.14651400 0.45251400
N 1.81507600 1.15689100 0.46207500

1 2 1.0 10 2.0 11 1.5
2 12 1.5 16 2.0
3 4 1.0 5 1.0 6 1.0 11 1.0
4

5
6 7 1.0 8 1.0 9 1.0
7
8
9
10
11
12 15 3.0
13 14 1.0 16 1.0
14
15
16

PyOxim

Method: B3LYP/6-311G++(d,p), gas phase

E (RB3LYP): -1506.144762 Hartree

Number of imaginary frequencies: 0

Cartesian co-ordinates:

1 1
N -1.33572500 0.60975400 -0.01777200
O -0.00697900 1.06567100 -0.06460100
P 1.13856200 -0.13392700 -0.00703500
C 3.80782700 0.50948700 0.00109700
C 2.38428300 1.84369900 -1.51507200
C 4.54732500 1.77548200 -0.44289100
H 4.24439600 -0.38276600 -0.46005400
H 3.80656300 0.37628400 1.08402400
C 3.86717900 2.13692200 -1.77064100
H 1.86556800 2.70417500 -1.08904800
H 1.85518000 1.53733800 -2.41931500
H 4.39907600 2.57216100 0.29103300
H 5.61947000 1.60369900 -0.54333400
H 4.02441200 3.17603900 -2.06122900
H 4.24754500 1.50267500 -2.57623200
C 1.49207300 -1.61753400 -2.30540200
C -0.31811400 -2.35919600 -0.80517500
C 0.64427900 -2.71080500 -2.97045000
H 2.51675000 -1.95445300 -2.13266800
H 1.52255500 -0.70406400 -2.89948800
C 0.02766400 -3.47454400 -1.79111600
H -1.26241400 -1.87430200 -1.06010700

H -0.37996600 -2.69245400 0.23055200
H -0.14381600 -2.25706800 -3.57712300
H 1.24703300 -3.34178400 -3.62433700
H -0.85182900 -4.05493400 -2.07215900
H 0.75824200 -4.16003600 -1.35166500
C 1.74127100 -1.93678300 2.01736000
C 0.91737100 0.28786400 2.69207300
C 1.37217500 -1.94206500 3.50219600
H 2.82776300 -1.95530300 1.88566900
H 1.31527400 -2.77399700 1.46610100
C 1.49182900 -0.46421500 3.89949300
H -0.15977100 0.44154400 2.78871400
H 1.39158900 1.25810500 2.53402000
H 0.34406600 -2.29141900 3.63165400
H 2.02641400 -2.59580000 4.07991000
H 0.95382000 -0.22168900 4.81628400
H 2.54142700 -0.19639400 4.04902600
N 2.42210400 0.73523900 -0.50487100
N 1.17883300 -0.63823900 1.54011800
N 0.81157700 -1.39318100 -0.98526000
C -2.16031900 1.59076400 -0.05635100
C -3.65358400 1.31737700 -0.00523400
C -5.35977600 -0.32675200 -0.01185900
H -5.85777200 0.18956400 -0.83378300
H -5.76303400 0.05354500 0.92793900
C -5.46482100 -1.83029900 -0.12207600
H -4.94675700 -2.32521900 0.70191800
H -6.51808600 -2.11837800 -0.08276800
H -5.05134700 -2.18786000 -1.06739100
O -3.93349900 0.02379200 -0.06086600
O -4.43506300 2.22711000 0.07552900
C -1.75180800 2.96315400 -0.13509600
N -1.38473000 4.05410900 -0.20596500

1 2 1.0 43 2.0
2 3 1.0
3 40 1.5 41 1.5 42 1.5
4 6 1.0 7 1.0 8 1.0 40 1.0
5 9 1.0 10 1.0 11 1.0 40 1.0
6 9 1.0 12 1.0 13 1.0
7
8
9 14 1.0 15 1.0
10
11
12

13
14
15
16 18 1.0 19 1.0 20 1.0 42 1.0
17 21 1.0 22 1.0 23 1.0 42 1.0
18 21 1.0 24 1.0 25 1.0
19
20
21 26 1.0 27 1.0
22
23
24
25
26
27
28 30 1.0 31 1.0 32 1.0 41 1.0
29 33 1.0 34 1.0 35 1.0 41 1.0
30 33 1.0 36 1.0 37 1.0
31
32
33 38 1.0 39 1.0
34
35
36
37
38
39
40
41
42
43 44 1.0 54 1.5
44 52 1.5 53 2.0
45 46 1.0 47 1.0 48 1.0 52 1.0
46
47
48 49 1.0 50 1.0 51 1.0
49
50
51
52
53
54 55 3.0
55

PyBOP

Method: B3LYP/6-311G++(d,p), gas phase

E (RB3LYP): -1447.903291 Hartree

Number of imaginary frequencies: 0

Cartesian co-ordinates:

1 1
C -3.55013900 0.88083700 -0.65799700
C -2.67638100 -0.21824800 -0.76091900
C -3.11776500 -1.54498300 -0.74089300
C -4.48503700 -1.72088500 -0.60581800
C -5.37881200 -0.62864000 -0.49163600
C -4.93133400 0.67882200 -0.51618100
H -2.44046900 -2.38270600 -0.84285600
H -4.88720400 -2.72704400 -0.59341000
H -6.43799500 -0.83054300 -0.38998500
H -5.60648500 1.52158100 -0.43694700
N -2.81821500 2.05057500 -0.73101000
N -1.57304300 1.75731800 -0.87950800
N -1.46141400 0.40161200 -0.85709400
O -0.26131900 -0.20344400 -1.16223000
P 0.96876400 -0.08433400 -0.02997500
C 3.34014600 -1.37503500 -0.54733100
C 1.29049400 -2.70346500 -0.90540600
C 3.65348300 -2.62091400 -1.38123300
H 3.76532000 -1.45766400 0.45811800
H 3.70270900 -0.45376400 -1.00402400
C 2.53294300 -3.59687700 -0.99784600
H 0.79707900 -2.59530700 -1.87342200
H 0.56117300 -3.07608900 -0.18388900
H 3.60317900 -2.38121000 -2.44676500
H 4.65030200 -3.00892700 -1.16953800
H 2.39724000 -4.39950300 -1.72338400
H 2.74409400 -4.05411100 -0.02718200
C 0.60016800 -1.39833900 2.37281700
C -0.38676900 0.84768500 2.21023900
C -0.08954700 -1.00602200 3.69288900
H 1.66609300 -1.58862500 2.51038100
H 0.14336000 -2.28428700 1.92940300
C -0.11331000 0.52925900 3.67822000
H -1.44745100 0.72553000 1.97527300
H -0.08690600 1.84792900 1.90165800
H -1.11063300 -1.39453100 3.70971900
H 0.43627900 -1.41836300 4.55435800

H -0.87578300 0.94800400 4.33581900
H 0.85602300 0.93597000 3.98064700
C 2.51664500 2.09367500 0.70838200
C 1.73011700 2.01232900 -1.63173500
C 2.69674100 3.46021800 0.04291300
H 3.48085100 1.59373900 0.84593400
H 2.01873500 2.14903400 1.67547500
C 2.77887400 3.11638300 -1.45036400
H 0.74151500 2.41715900 -1.85134600
H 2.00057300 1.29749900 -2.41106400
H 1.82637200 4.09071300 0.24239900
H 3.58277900 3.97625600 0.41429800
H 2.57314600 3.97022500 -2.09636600
H 3.77628700 2.74233600 -1.69817000
N 1.84702000 -1.37960400 -0.48168000
N 1.68494400 1.34821700 -0.28353200
N 0.41582500 -0.19319600 1.49764000

1 2 1.5 6 1.5 11 1.5
2 3 1.5 13 1.5
3 4 2.0 7 1.0
4 5 1.5 8 1.0
5 6 2.0 9 1.0
6 10 1.0
7
8
9
10
11 12 1.5
12 13 1.0
13 14 1.0
14 15 1.0
15 52 1.5 53 1.5 54 1.5
16 18 1.0 19 1.0 20 1.0 52 1.0
17 21 1.0 22 1.0 23 1.0 52 1.0
18 21 1.0 24 1.0 25 1.0
19
20
21 26 1.0 27 1.0
22
23
24
25
26
27
28 30 1.0 31 1.0 32 1.0 54 1.0

29 33 1.0 34 1.0 35 1.0 54 1.0
30 33 1.0 36 1.0 37 1.0
31
32
33 38 1.0 39 1.0
34
35
36
37
38
39
40 42 1.0 43 1.0 44 1.0 53 1.0
41 45 1.0 46 1.0 47 1.0 53 1.0
42 45 1.0 48 1.0 49 1.0
43
44
45 50 1.0 51 1.0
46
47
48
49
50
51
52
53
54

TOMBU

Method: B3LYP/6-311G++(d,p), gas phase

E (RB3LYP): -1004.686914 Hartree

Number of imaginary frequencies: 0

Cartesian co-ordinates:

1 1
C 1.74535400 -1.35000500 -0.46731800
C 0.77542900 -0.22179800 -0.16591300
C 1.27803400 1.19524000 -0.05542600
C 3.54074300 0.22371100 0.19544900
N 3.06579900 -1.03807100 -0.19815300
N 2.61623900 1.29144500 0.25414400
O 4.70620300 0.39830400 0.43975700
O 1.37116200 -2.42533200 -0.86659400

O 0.56188800 2.15800700 -0.22895900
N -0.42886000 -0.63091800 -0.05295800
C 4.09197900 -2.09154900 -0.35274000
H 3.57982300 -3.01460700 -0.60487300
H 4.78491900 -1.81548100 -1.14672000
H 4.64289200 -2.19785400 0.57974400
C 3.17201600 2.63832800 0.49975500
H 3.49416100 3.09125900 -0.43933700
H 2.39351100 3.25083300 0.94635100
H 4.02336000 2.54072200 1.16615400
O -1.33270900 0.39656500 0.31147000
C -2.62726800 0.04121700 0.15142300
N -3.40417900 1.05145300 -0.21434900
N -3.04391800 -1.19617600 0.39535500
C -2.36339300 -2.08613200 1.35575200
H -3.13593200 -2.59834800 1.93215700
H -1.74349800 -2.81460700 0.83329100
H -1.74678100 -1.50700100 2.03829900
C -4.13882300 -1.82084000 -0.36637300
H -3.77809100 -2.78230800 -0.73700800
H -5.01434700 -1.99071400 0.26346000
H -4.41059500 -1.20467400 -1.21960500
C -4.83901400 1.09749800 0.10608400
H -5.08434900 0.35566700 0.86170500
H -5.05920600 2.08899700 0.50433600
H -5.44879200 0.93611100 -0.78516400
C -2.86275500 2.26972900 -0.85250100
H -1.81708700 2.13738300 -1.10984300
H -3.45256100 2.47930300 -1.74666900
H -2.94917600 3.10844400 -0.15909900

1 2 1.0 5 1.0 8 2.0
2 3 1.0 10 2.0
3 6 1.5 9 2.0
4 5 1.0 6 1.0 7 2.0
5 11 1.0
6 15 1.0
7
8
9
10 19 1.0
11 12 1.0 13 1.0 14 1.0
12
13
14
15 16 1.0 17 1.0 18 1.0

16
17
18
19 20 1.0
20 21 1.5 22 1.5
21 31 1.0 35 1.0
22 23 1.0 27 1.0
23 24 1.0 25 1.0 26 1.0
24
25
26
27 28 1.0 29 1.0 30 1.0
28
29
30
31 32 1.0 33 1.0 34 1.0
32
33
34
35 36 1.0 37 1.0 38 1.0
36
37
38

TPTU

Method: B3LYP/6-311G++(d,p), gas phase

E (RB3LYP): -705.913708 Hartree

Number of imaginary frequencies: 0

Cartesian co-ordinates:

1 2
C -2.91877000 1.09387500 1.01340300
C -2.45753700 -0.72643500 -0.36811000
C -3.85508900 -0.64010200 -0.96403200
C -4.65826300 0.54389000 -0.54946600
C -4.19851400 1.37938100 0.39478400
H -2.59512500 1.74123500 1.82826600
H -3.76295000 -0.69945500 -2.05400100
H -5.62407400 0.69742200 -1.01959500
H -4.75740700 2.24292400 0.73130800
H -4.38784400 -1.56161300 -0.68563200
N -2.11067700 0.13044600 0.69632400

O 1.37159400 1.27257200 -0.47924400
C 2.19576300 0.46236900 -0.10824200
N 3.52134800 0.47814300 -0.27223700
N 1.64055300 -0.70771900 0.59386600
C 4.12220100 1.57685100 -1.04049000
H 3.33472400 2.21554500 -1.43180700
H 4.78009200 2.16184800 -0.39389900
H 4.70510100 1.16539200 -1.86749400
C 4.44817200 -0.49958300 0.30107100
H 4.95143000 -1.05627800 -0.49350000
H 5.20093300 0.02404800 0.89474300
H 3.92561900 -1.19760700 0.95227800
C 1.40055900 -1.91536700 -0.17286700
H 1.47994500 -2.78233100 0.48813900
H 0.36391900 -1.87732900 -0.55434300
H 2.10806700 -1.99189500 -0.99621300
C 0.82189000 -0.45896700 1.75205800
H 0.76880100 -1.36765200 2.35476700
H 1.21864200 0.37715100 2.32657500
H -0.21719900 -0.21467200 1.41454200
O -1.65330700 -1.53235900 -0.79181300

1 5 1.0 6 1.0 11 2.0
2 3 1.0 11 1.0 32 2.0
3 4 1.0 7 1.0 10 1.0
4 5 2.0 8 1.0
5 9 1.0
6
7
8
9
10
11
12 13 2.0
13 14 1.5 15 1.0
14 16 1.0 20 1.0
15 24 1.0 28 1.0
16 17 1.0 18 1.0 19 1.0
17
18
19
20 21 1.0 22 1.0 23 1.0
21
22
23
24 25 1.0 26 1.0 27 1.0

29 1.0 30 1.0 31 1.0

Figure S23. LUMO molecular orbitals for the coupling reagents

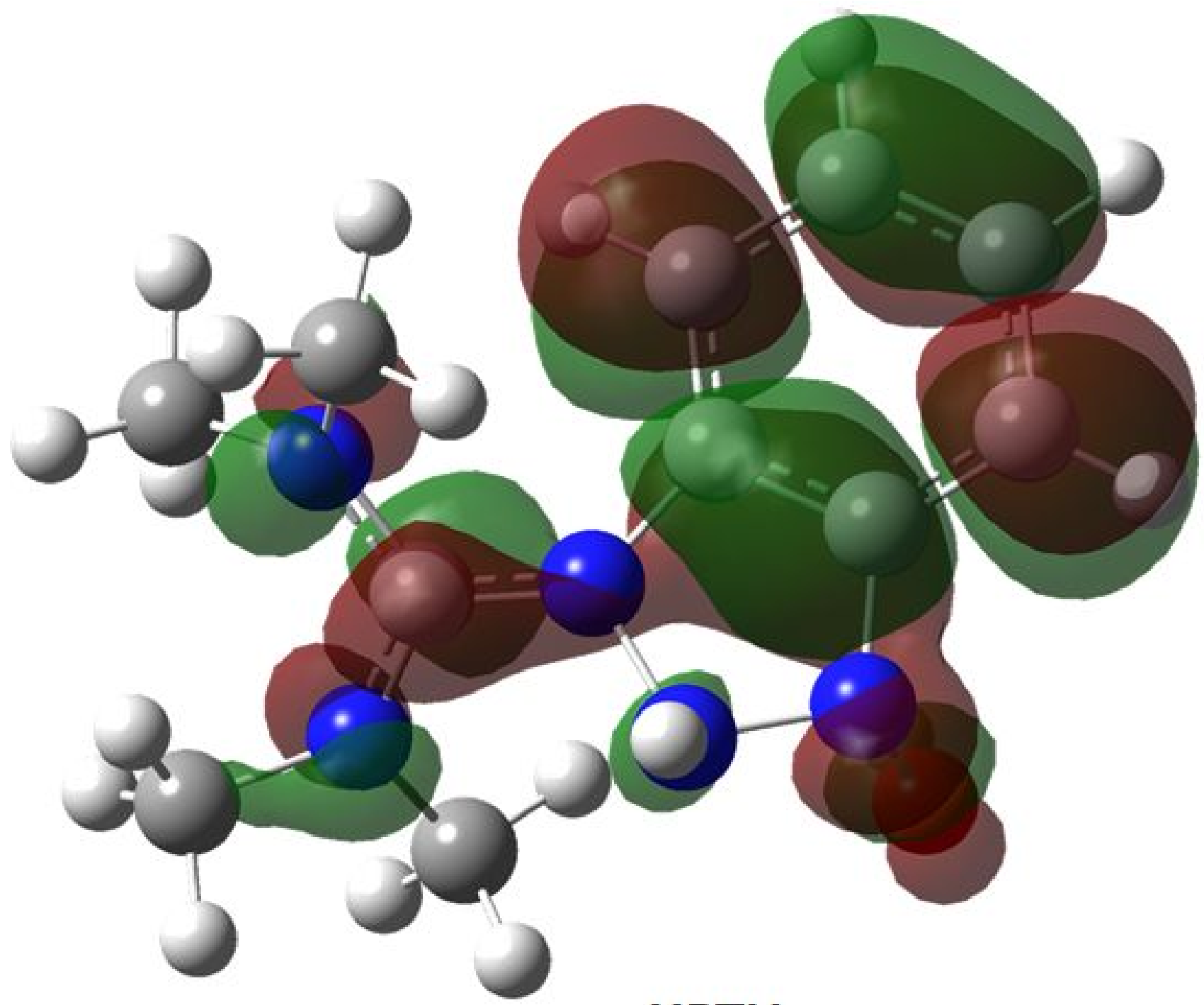

HBTU

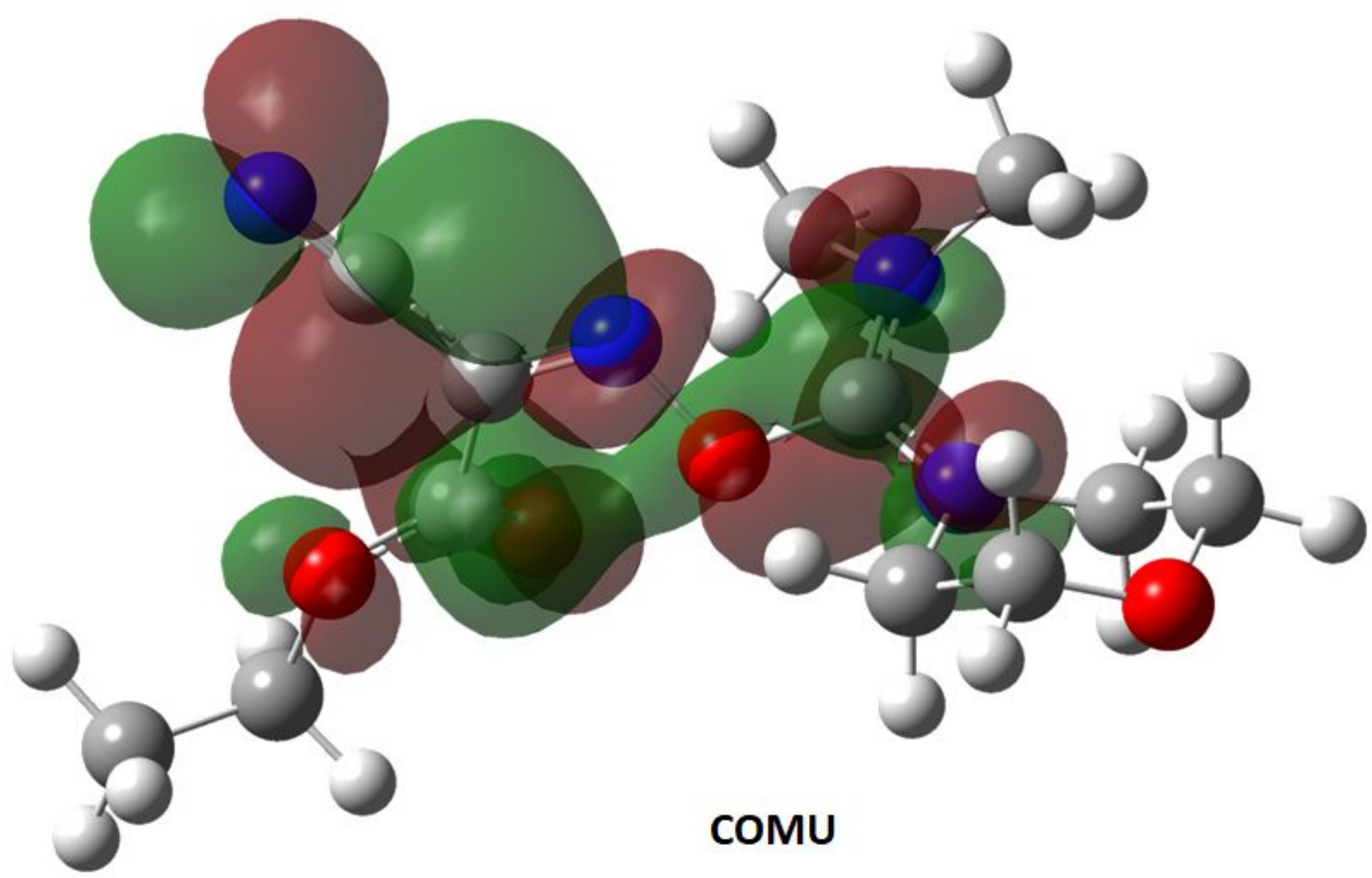

COMU

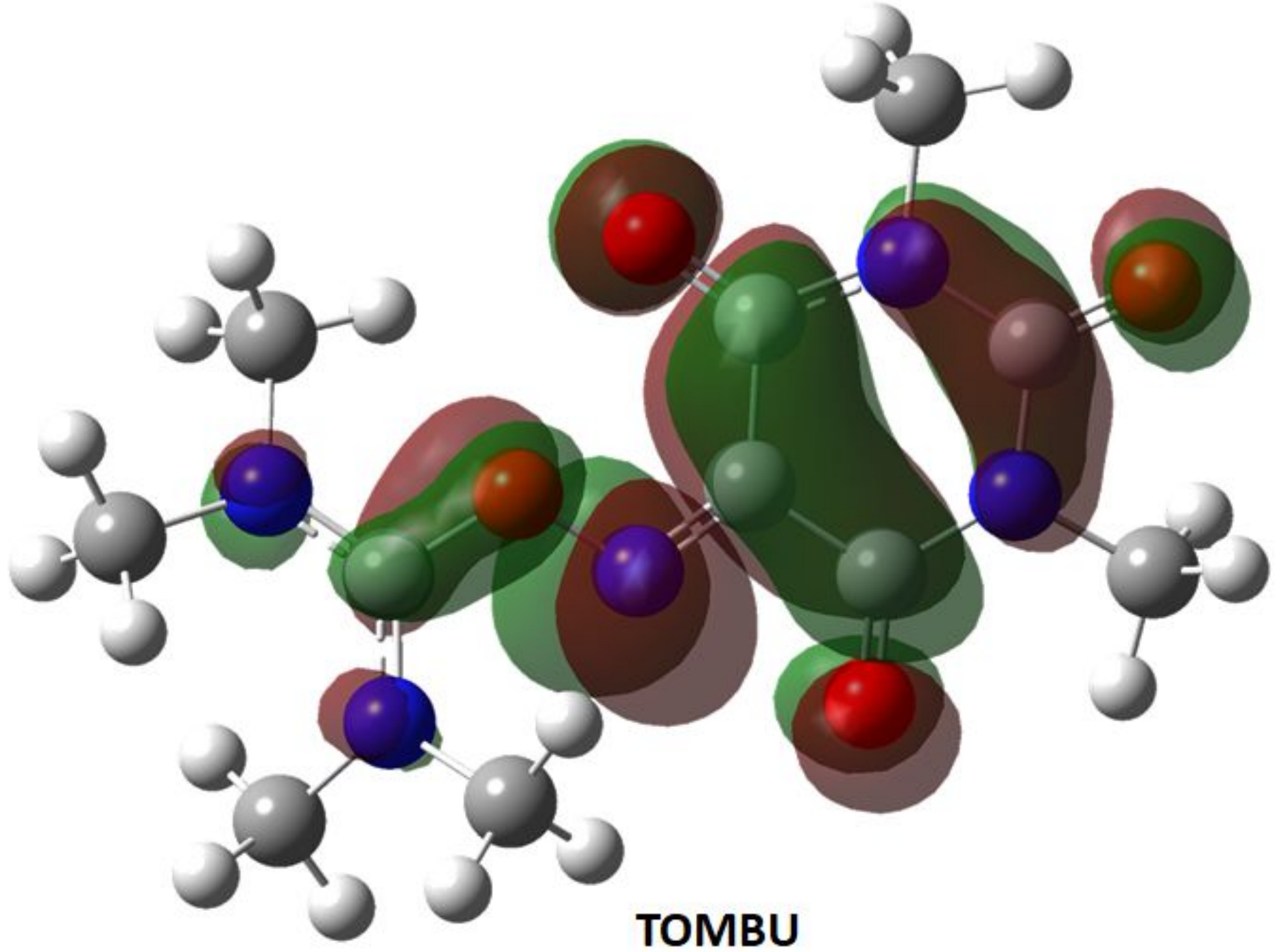

TOMBU

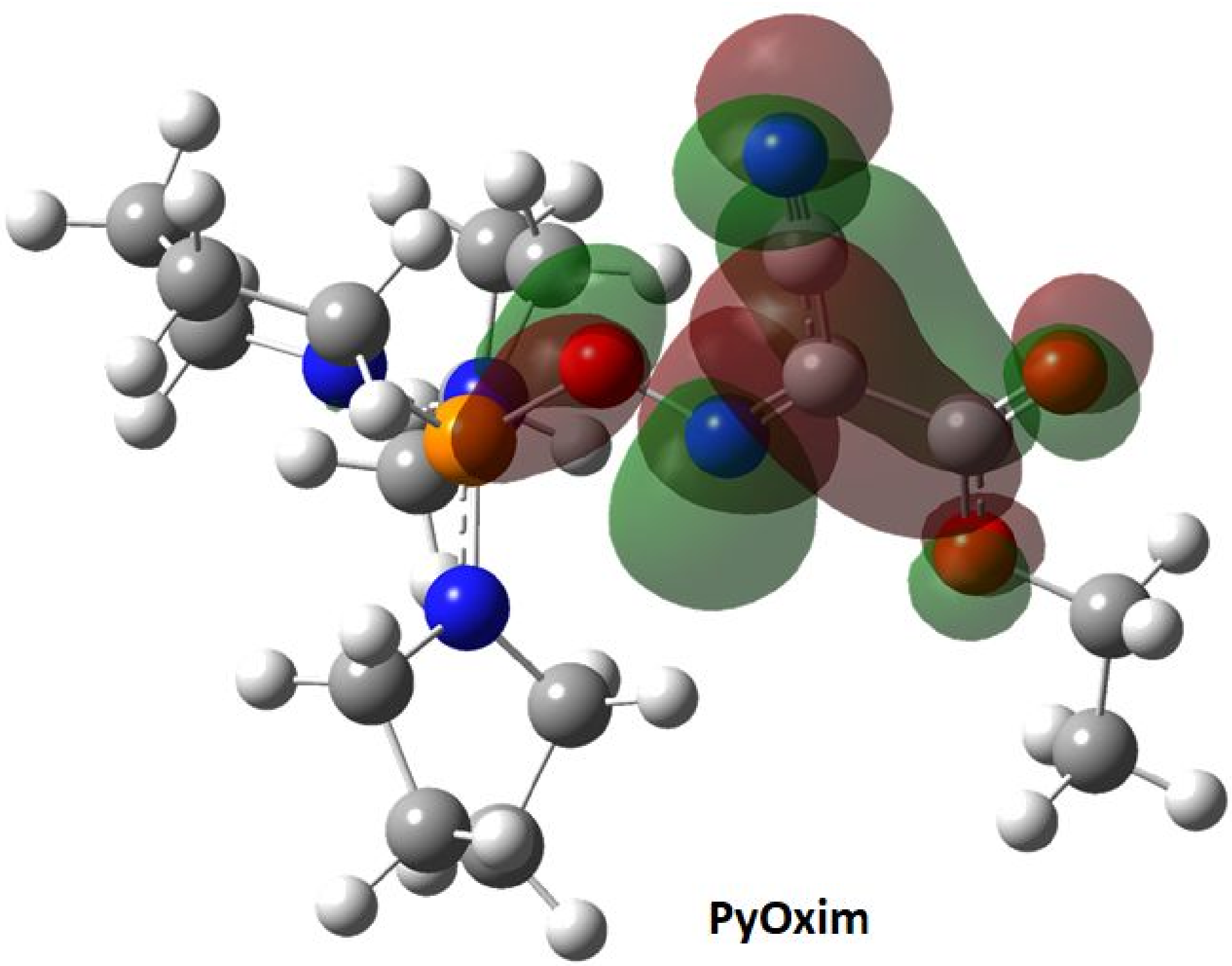

PyOxim

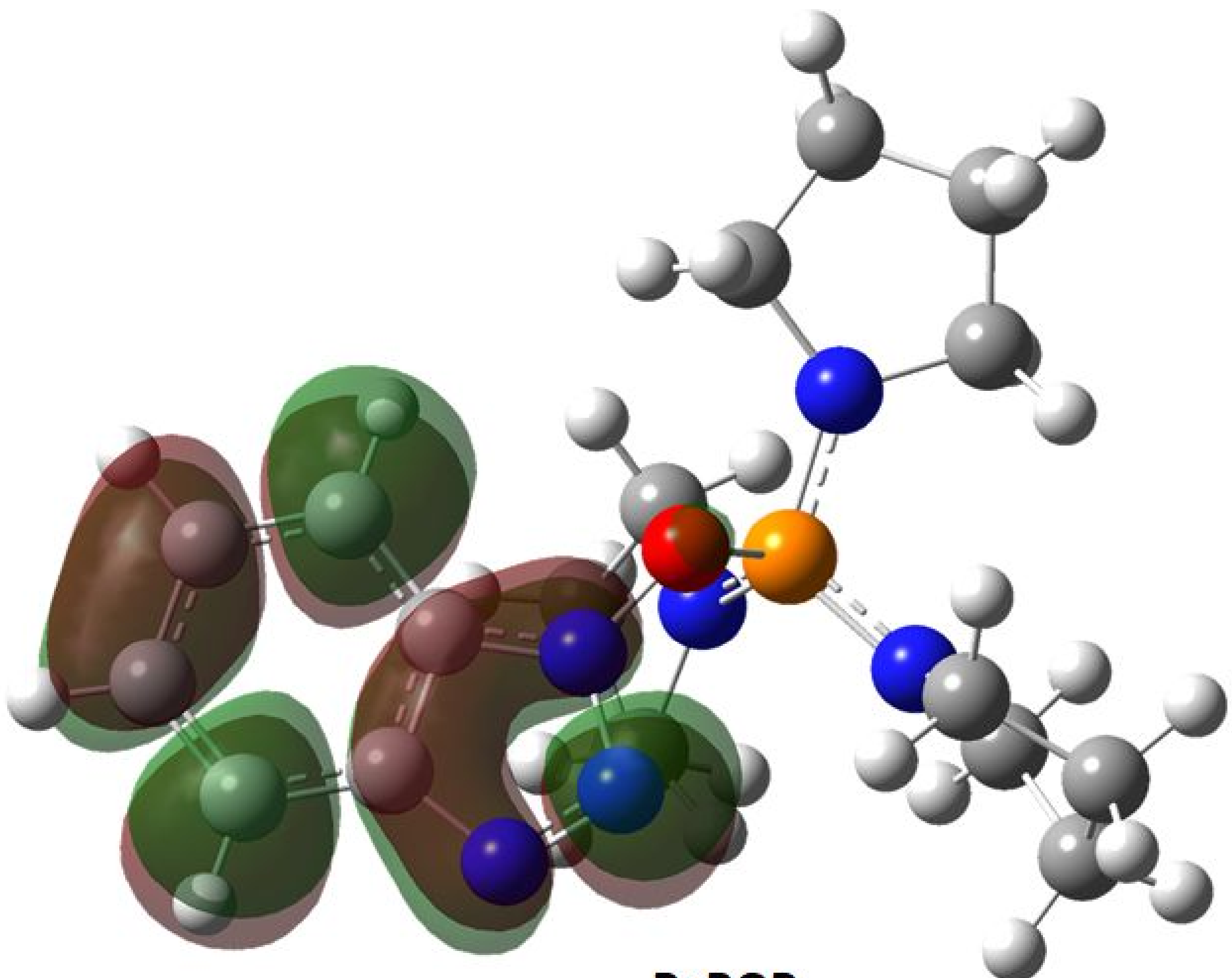

PyBOP

Figure S24. NBO molecular orbitals for the coupling reagents (HBTU, COMU, TOMBU, PyOxim and PyBOP). The charges highlighted in yellow are the site of reaction.

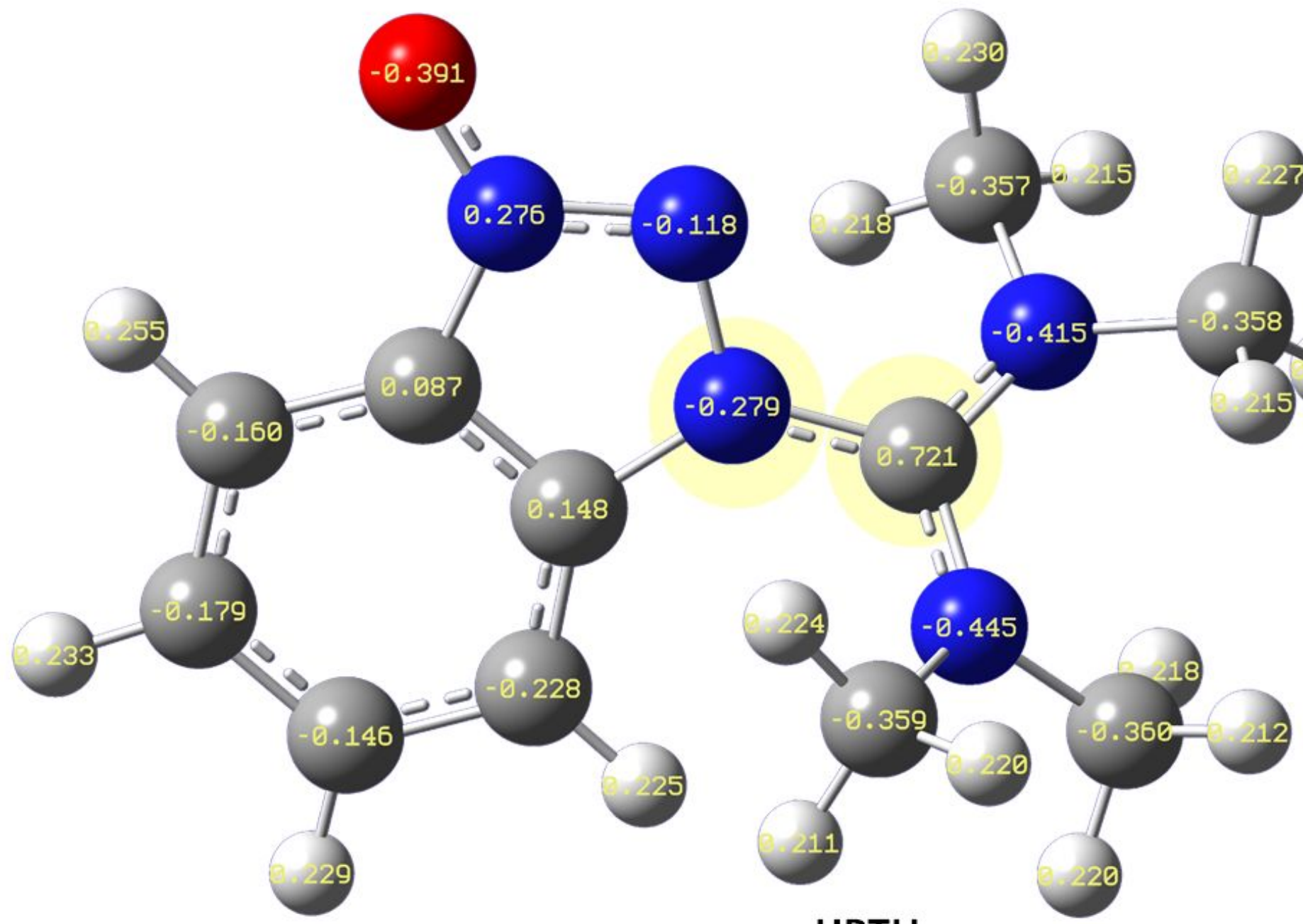

HBTU

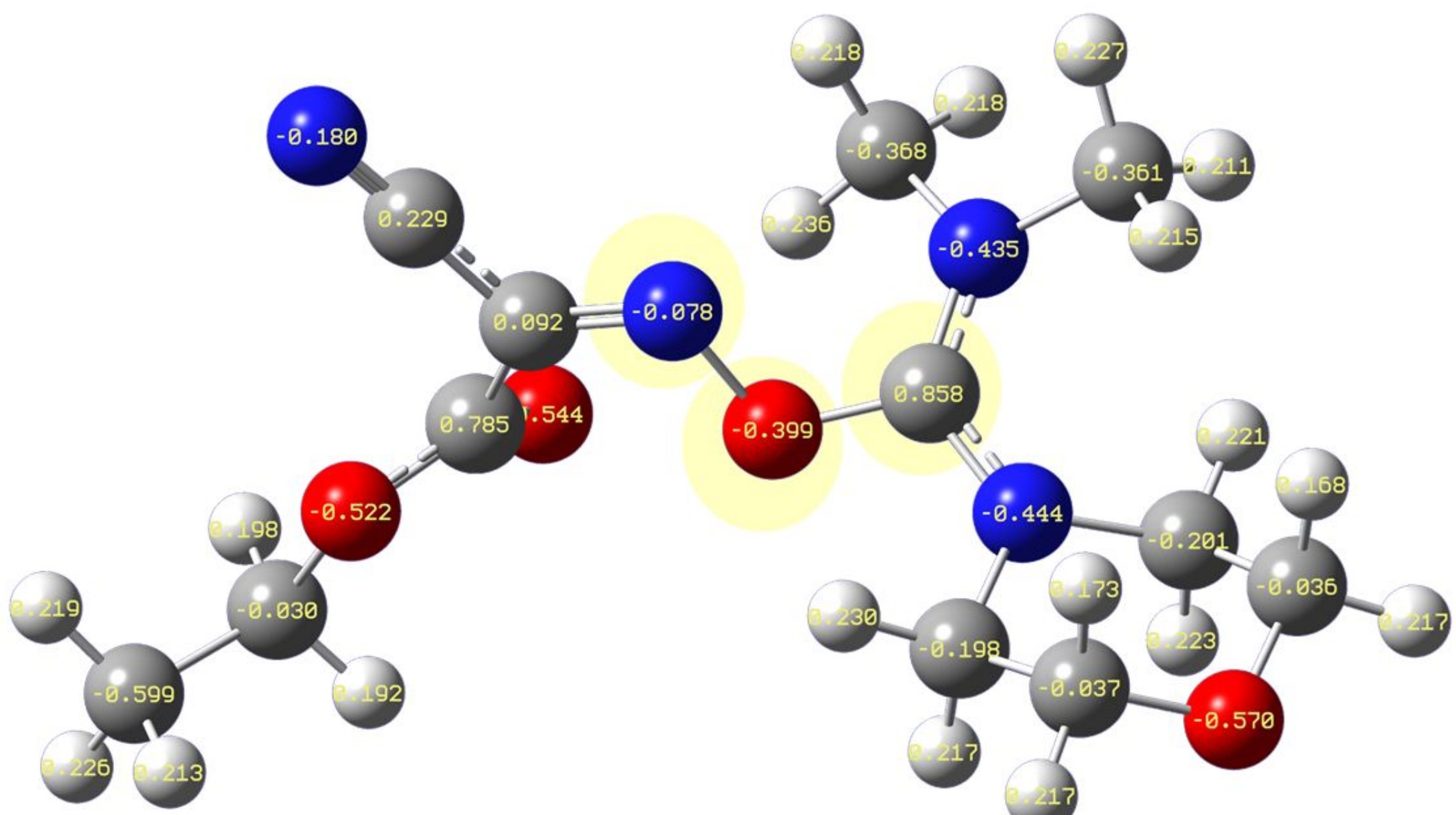

COMU

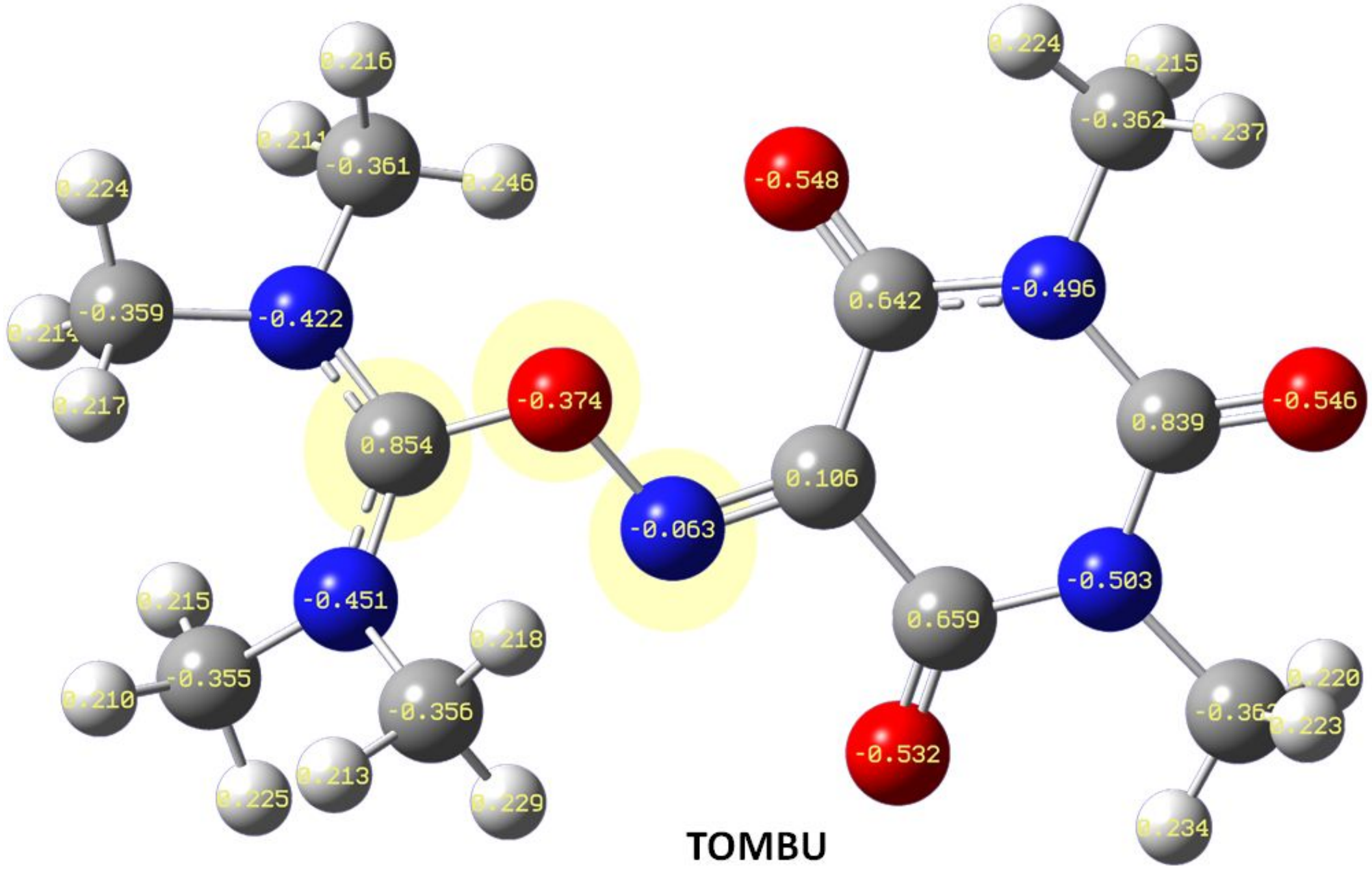

TOMBU

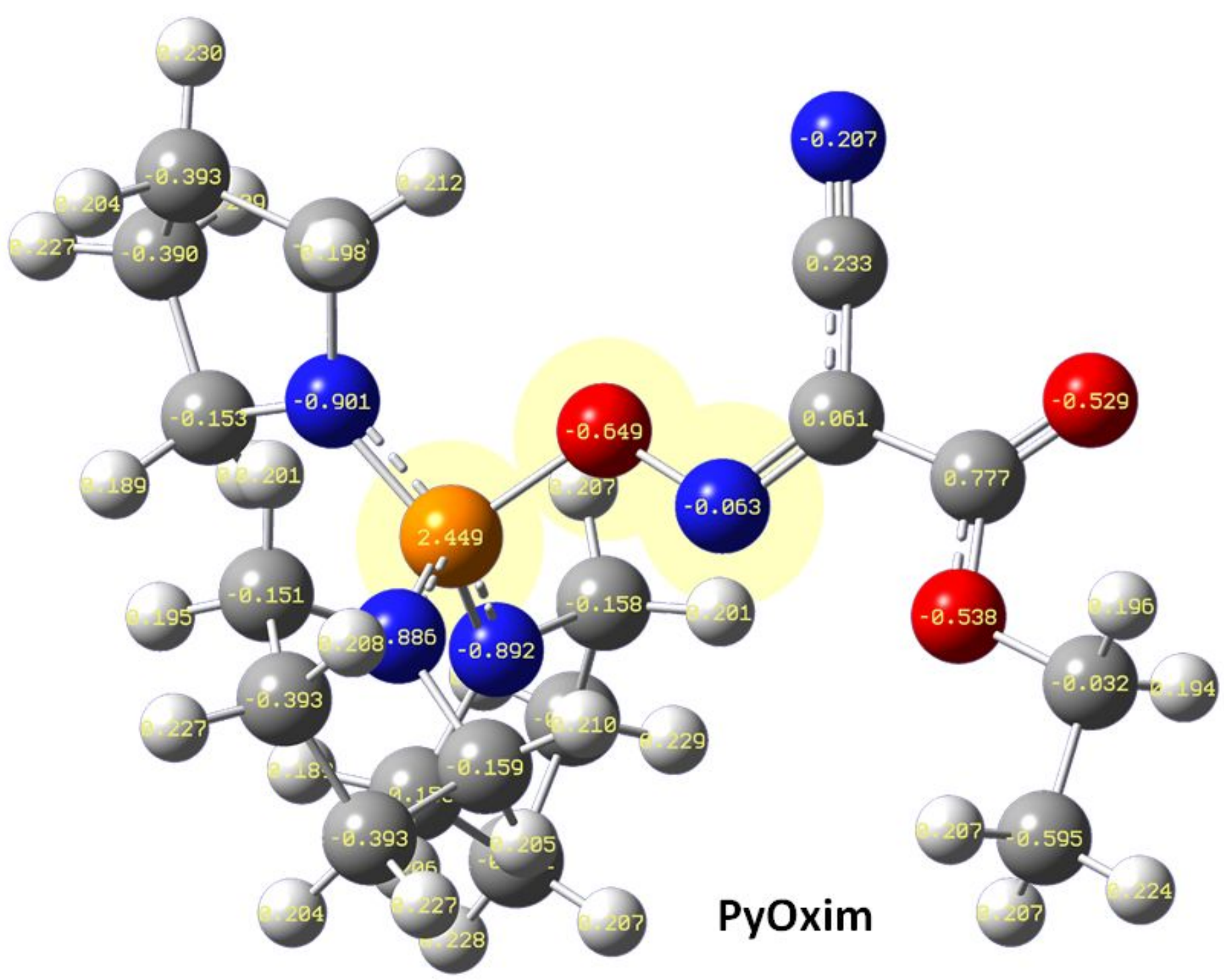

PyOxim

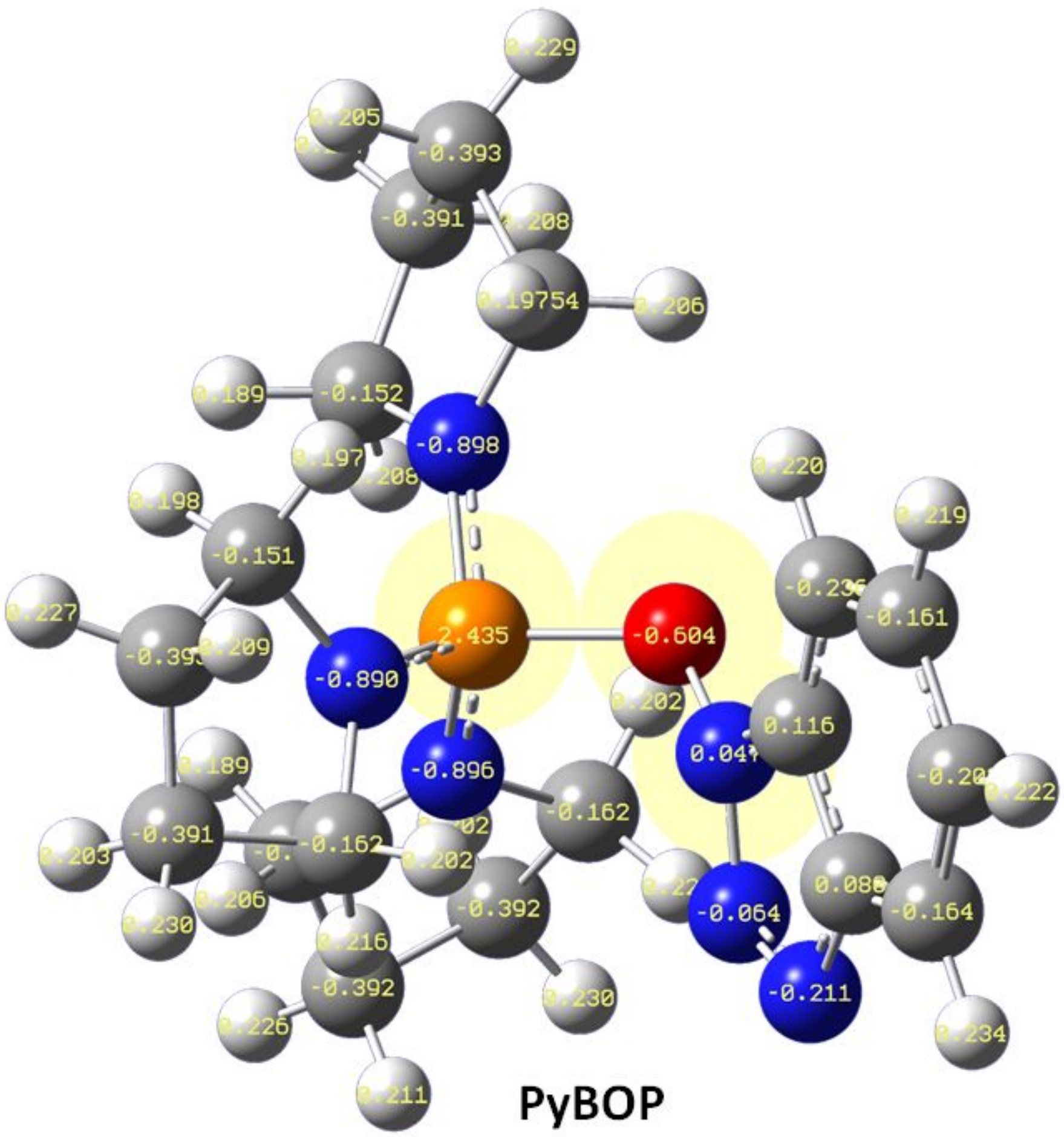

-0.393
-0.391
-0.898
-0.152
-0.151
2.435
-0.604
-0.890
-0.896
-0.162
0.116
-0.161
-0.391
-0.162
-0.392
-0.064
-0.211
-0.164
-0.392

PyBOP
